# Crystal structure of the HMGA AT-hook 1 domain bound to the minor groove of AT-rich DNA and inhibition by antikinetoplastid drugs

**DOI:** 10.1038/s41598-024-77522-3

**Published:** 2024-10-30

**Authors:** J. Jonathan Nué-Martinez, Marta Maturana, Laura Lagartera, Juan-Antonio Rodríguez-Gutiérrez, Roeland Boer, J. Lourdes Campos, Núria Saperas, Christophe Dardonville

**Affiliations:** 1 Instituto de Química Médica, IQM–CSIC, Madrid, 28006 Spain; 2 Departament d’Enginyeria Química, EEBE, Universitat Politècnica de Catalunya, Barcelona, 08019 Spain; 3 ALBA Synchrotron Light Source, Experiments división, Cerdanyola del Valles, 08290 Barcelona, Spain

**Keywords:** High mobility group (HMG) protein, HMGA AT-hook 1 domain, AT-hook 1 binding inhibitor, DNA minor groove binder, kinetoplastid parasite, crystal structure., DNA, Parasitic infection, Target identification, DNA-binding proteins, Biophysical chemistry

## Abstract

High mobility group (HMG) proteins are intrinsically disordered nuclear non-histone chromosomal proteins that play an essential role in many biological processes by regulating the expression of numerous genes in eukaryote cells. HMGA proteins contain three DNA binding motifs, the “AT-hooks”, that bind preferentially to AT-rich sequences in the minor groove of B-form DNA. Understanding the interactions of AT-hook domains with DNA is very relevant from a medical point of view because HMGA proteins are involved in different conditions including cancer and parasitic diseases. We present here the first crystal structure (1.40 Å resolution) of the HMGA AT-hook 1 domain, bound to the minor groove of AT-rich DNA. In contrast to AT-hook 3 which bends DNA and shows a larger minor groove widening, AT-hook 1 binds neighbouring DNA molecules and displays moderate widening of DNA upon binding. The binding affinity and thermodynamics of binding were studied in solution with surface plasmon resonance (SPR)-biosensor and isothermal titration calorimetry (ITC) experiments. AT-hook 1 forms an entropy-driven 2:1 complex with (TTAA)_2_-containing DNA with relatively slow kinetics of association/dissociation. We show that *N*-phenylbenzamide-derived antikinetoplastid compounds (**1**–**3**) bind strongly and specifically to the minor groove of AT-DNA and compete with AT-hook 1 for binding. The central core of the molecule is the basis for the observed sequence selectivity of these compounds. These findings provide clues regarding a possible mode of action of DNA minor groove binding compounds that are relevant to major neglected tropical diseases such as leishmaniasis and trypanosomiasis.

**Supplementary Information:**

The online version contains supplementary material available at 10.1038/s41598-024-77522-3.

## Introduction

Leishmaniasis, American (Chagas’ disease) and African trypanosomiases are zoonosis caused by kinetoplastid parasites of the Trypanosomatidae family (i.e., *Leishmania*, *Trypanosoma cruzi* and *T. brucei*, respectively), which are mainly transmitted by insect vectors in tropical and subtropical regions around the globe. Nowadays, leishmaniasis and Chagas’ disease are spreading outside the endemic regions due to climate and demographic changes, human behavioural aspects and co-infections^1,2^. Existing therapies for treating kinetoplastid diseases are inappropriate due to several factors including low therapeutic indexes, high toxicities associated with unacceptable side-effects, prices that are unaffordable for most patients in the most affected countries, and the difficulty of treatment compliance due to complex protocols^3^. The latter leads to the appearance of drug resistant strains resulting in loss of treatment efficacy. Hence, the identification of new therapeutic targets specific to these parasites, and the discovery of new drugs acting on these targets, is an important goal towards the development of new antiprotozoal drugs for human and animal health^4^.

**Fig. 1:**
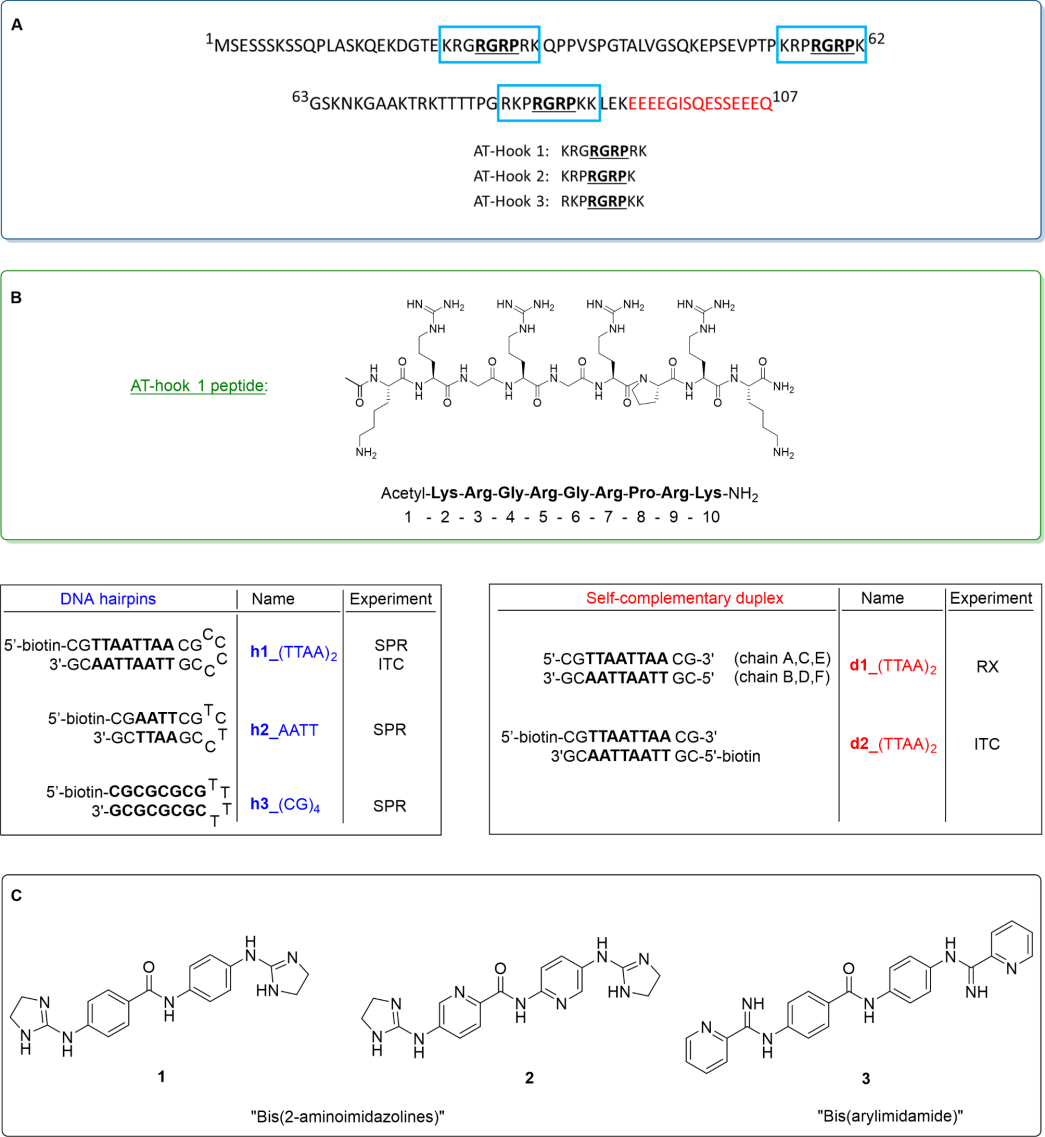
(**A**) Primary structure of the human HMGA1a protein highlighting the three AT-hook binding motifs. The conserved RGRP core sequence is shown in bold and underlined. In red, the acidic C-terminal tail. (**B**) Sequences of the AT-hook 1 peptide and the oligonucleotide duplexes used in the crystallographic and biophysical studies. (**C**) Structures of compounds **1**–**3** used in this study.

Trypanosomatid parasites present remarkable features, such as a single mitochondrion that contains an enlarged region, termed kinetoplast, which harbours the mitochondrial DNA (kDNA) organized as a tightly packed disk of intercalating maxicircles (up to 23 kb) with thousands of minicircles of approximately 1 kb^5,6^. kDNA, which is particularly rich in AT DNA sequences^7^, is a good target of dicationic minor groove binders (MGBs) such as bis(2-aminoimidazolines) **1**–**2** or bis(arylimidamide) **3** (Fig. 1)^8^. The selectivity of dicationic MGBs towards kDNA is favoured by the accumulation of this class of compounds in the mitochondrion of the parasites, driven by the electrostatic transmembrane potential (negative inside). We have shown that the bis(2-aminoimidazoline) class of MGBs is very effective against *T. brucei*^9–11^, whereas bis(arylimidamides) are more active against the intracellular parasites *T. cruzi* and *Leishmania*^8^. Compound **1**, for instance, acts specifically on the integrity of the kinetoplast by altering the structure and replication of *T. brucei* kDNA and possibly by interfering with ‘high mobility group’ (HMG) box-containing proteins essential for kDNA function^12^. This could conceivably occur by inhibition of the mitochondrial HMG-box protein TbKAP6, which is essential for kDNA replication and maintenance^13^.

The HMG protein family is one of the largest groups of nuclear non-histone chromosomal proteins. HMG proteins are involved in numerous biological processes such as cell growth, proliferation, differentiation and apoptosis by regulating the expression of numerous genes through alteration of chromatin architecture^14,15^. These proteins, which have a high content of charged amino acids, are classified into three subfamilies (i.e., HMGA, HMGB and HMGN), each possessing an exclusive and distinctive DNA binding motif: the ‘AT-hook’ (HMGA family, Fig. 1A), the ‘HMG-box’ (HMGB family), and the ‘nucleosomal binding domain’ (HMGN family)^14^. HMGA are intrinsically disordered proteins which contain three AT-hooks that bind preferentially to AT-rich sequences in the minor groove of B-form DNA^16^. The AT-hook motif is formed by a short stretch of amino acids containing a conserved core sequence, Arg-Gly-Arg-Pro (R-G-R-P), flanked by other positively charged amino acids (Fig. 1A)^17^. Free in solution, AT-hooks barely show secondary structure before interaction with other macromolecules, which may explain the shortage of crystallographic structural data available on the interaction of AT-hooks with DNA.

In previous work, we reported the crystal structure of the complex of a DNA oligonucleotide d(CGAATTAATTCG)_2_ with the third AT-hook (DBD3 in reference^18^) of the HMGA1 protein^19^, which until now was the only solved crystallographic structure of a DNA complex with an AT-hook. In the present study, we elucidate the crystal structure of the complex of AT-hook 1 (KRGRGRPRK) with the DNA oligonucleotide d(CGTTAATTAACG)_2_ at 1.40 Å resolution (Fig. 1B). The strength and thermodynamics of binding of the AT-hook 1 peptide were also characterized using surface plasmon resonance (SPR)–biosensor and isothermal titration calorimetry (ITC) experiments.

Eventually, we sought to determine whether the antikinetoplastid drugs **1**–**3** could inhibit the binding of AT-hook 1 to DNA. Such inhibition capacity is relevant from a “mode of action” point of view because AT-hook proteins such as LamAT-Y, which is expressed in both promastigote and amastigote stages of *L. major*, have AT-hook domains functionally equivalent to those of HMGA1a^20^. The critical role of AT-hook proteins for the normal biology of *Leishmania* parasites is thought to involve several processes including gene expression, DNA replication and DNA repair^20^. Indeed, SPR and ITC experiments showed that compounds **1**–**3** bind strongly and specifically to AT-DNA sequences and compete with AT-hook 1 for binding in the minor groove of DNA. This inhibitory capacity is relevant not only for antiparasitic chemotherapy, but also in the field of cancer where HMGA proteins are involved in neoplastic transformation and tumour progression. Notably, the overexpression of HMGA has been found in a variety of cancers, where it influences the transcription of several genes crucial for cancer development^21,22^.

## Results

### Crystallographic structure of the AT-hook 1-DNA complex

**Fig. 2:**
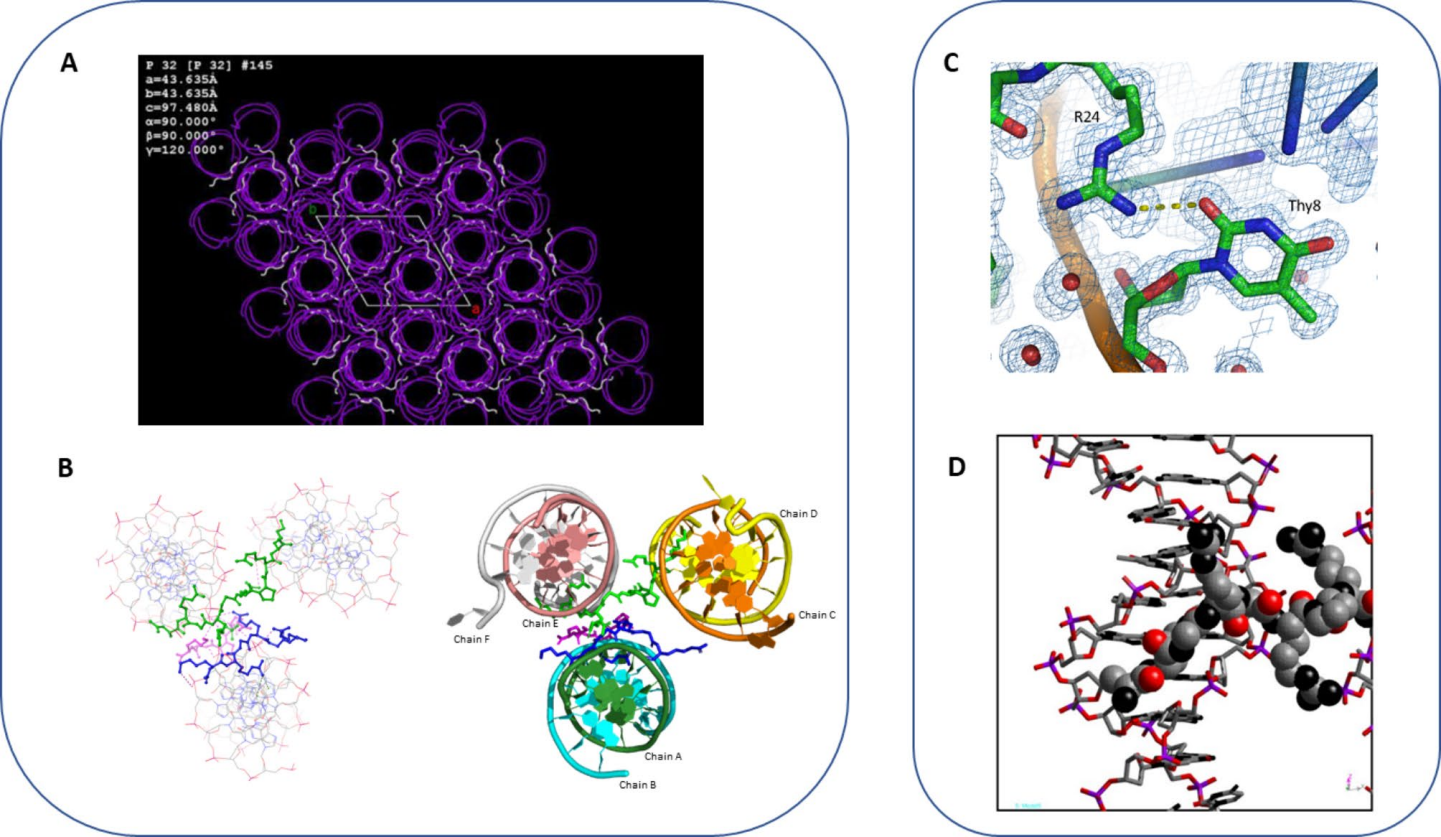
*Left panel*: (**A**) Top view of the asymmetric unit and crystal packing of the complex (PDB ID: 8CPG). Peptides are shown as white filaments. (**B**) A section of the structure. The terminal bases of the dodecamers do not form Watson-Crick base pairs; instead, the terminal guanines occupy an external position and form N2-N3 hydrogen bonds with the guanines from a neighbour stacked duplex. The cytosines occupy external positions; some of them are highly disordered and do not appear in the electron density map. The three AT-hook 1 peptides are highlighted in green (G), blue (H), and pink (I). *Right panel*: Interaction of the DNA duplexes with the AT-hook 1 peptides present in the crystal. (**C**) Electron density map (2Fo-Fc at 1sigma level) of a segment of AT-hook 1 in the minor groove of DNA. Hydrogen bond between main chain peptide NH atom (Arg 24 from HMGA = Arg 3 from AT-hook 1 peptide G shown in Fig. 1B) and thymine 8 of chain F is indicated as yellow dashed line. (**D**) Peptide G is fully visible in the structure. It has the expected conformation for an AT-hook 1 into the minor groove of AT-rich DNA (only chains E-F are shown).

The crystal structure of the complex of AT-hook 1 (KRGRGRPRK) with the DNA oligonucleotide d(CGTTAATTAACG)_2_ has been solved at 1.40 Å resolution (PDB-ID: **8CPG**). The asymmetric unit of the P32 crystal structure and packing is presented in Table 1; Fig. 2A. It is formed by three independent parallel DNA duplexes (AB, CD, and EF chains) and three AT-hook 1 peptide units (G, H and I). Their sequence is presented in Fig. 1B. Four Mg ions also contribute to stabilize the crystal structure.

The structure is very similar to that found in other dodecamers in the same space group^23^. It consists of three parallel columns of stacked dodecamer duplexes. A section of the structure is presented in Fig. 2B. The terminal bases of the dodecamers do not form Watson-Crick base pairs; instead, the terminal guanines occupy an external position and form N2-N3 hydrogen bonds with the guanines from a neighbour stacked duplex. The terminal cytosines occupy an external position and, in several cases, they are disordered and not visible in the crystallographic structure. As a result, the stacked columns are actually formed by the central decamer structure which has standard Watson-Crick base pairs. A detailed description of this type of trigonal structure has been presented elsewhere and we will not discuss it further^23^.

**Table 1:** Data collection and refinement statistics. Values in brackets correspond to the high-resolution shell.

Data collection	
Beamline	BL13-XALOC, ALBA
Wavelength (Å)	0.97925
Resolution range (Å)	37.79–1.40 (1.424-1.40)
Space group	P 32
Unit cell parameters (Å, °)	*a* = 43.63 *b* = 43.63 *c* = 97.48 α = 90.00 β = 90.00 γ = 120.00
Total reflections	342,471 (16148)
Unique reflections	40,811 (2030)
Multiplicity	8.4 (8.0)
Completeness (%)	99.8 (99.0)
Mean I/sigma(I)	18.6 (3.9)
Wilson B-factor	16.1
R-merge	0.067 (0.58)
Refinement statistics	
Reflections used in refinement	38,850
Reflections used for R-free	1975 (4.84%)
R-work	0.124
R-free	0.158
Number of non-hydrogen atoms	1966
Asymmetric unit (partially disordered)	3 DNA duplexes, 3 peptides, 4 Mg^+^
DNA	1426
Protein Atoms	169
Acetyl Atoms	6
Mg^2+^	4
RMS bonds (Å)	0.007
RMS angles (°)	1.58
Average B-factor, all atoms (Å^2^)	16.0
F_o_, F_c_ correlation	0.98
Water	361
PDB-ID	8CPG

^+^Statistics for the highest-resolution shell are shown in parentheses.

Our main interest is the analysis of the interaction of the DNA duplexes with the AT-hook 1 peptides present in the crystal. As mentioned above, there are three independent AT-hook 1 peptide molecules (G, H, I). Each of them interacts in a different way with DNA:

*Peptide I* is partially ordered. The only amino acids that are visible in the structure are GRPRK-NH_2_ (residues 6–10). As shown in Figure S5, this peptide does not interact with the grooves of the DNA duplexes. It stabilizes the crystal structure by ionic interactions with DNA phosphate groups of the AB and EF DNA duplexes.

*Peptide G* is fully visible in the structure. It has the conformation expected for an AT-hook; its R3-G4 region enters into the minor groove of the AT region of the EF oligonucleotide duplex (Fig. 2C and D and Supplementary Figures S2-S4). Also, the neighbouring K2 and R5 residues interact with EF phosphates and increase the stability of this AT-hook 1. The rest of the peptide extends away and shows ionic interactions with the CD oligonucleotide duplex. Thus, this peptide also has crosslinking features which stabilize the crystal structure.

*Peptide H* is partially ordered, the amino acid residues 1–7 (acetyl-KRGRGR) are visible in the structure. They show a similar AT-hook structure with the equivalent amino acids of Peptide G, but instead they interact with the AB DNA duplex.

In summary, two peptides and two oligonucleotide duplexes present clear AT hooks: peptide **G** with chains E-F and chains C-D, and peptide **H** with chains A-B. The other **I** peptide stabilizes A-B and E-F oligonucleotide duplexes by ionic interactions. The peptide-DNA interactions are shown in more detail in the Supplementary section (Figures S2–S6).

## DNA Binding analysis

### Characterization of AT-hook 1 DNA binding affinity, selectivity and thermodynamics

**Fig. 3:**
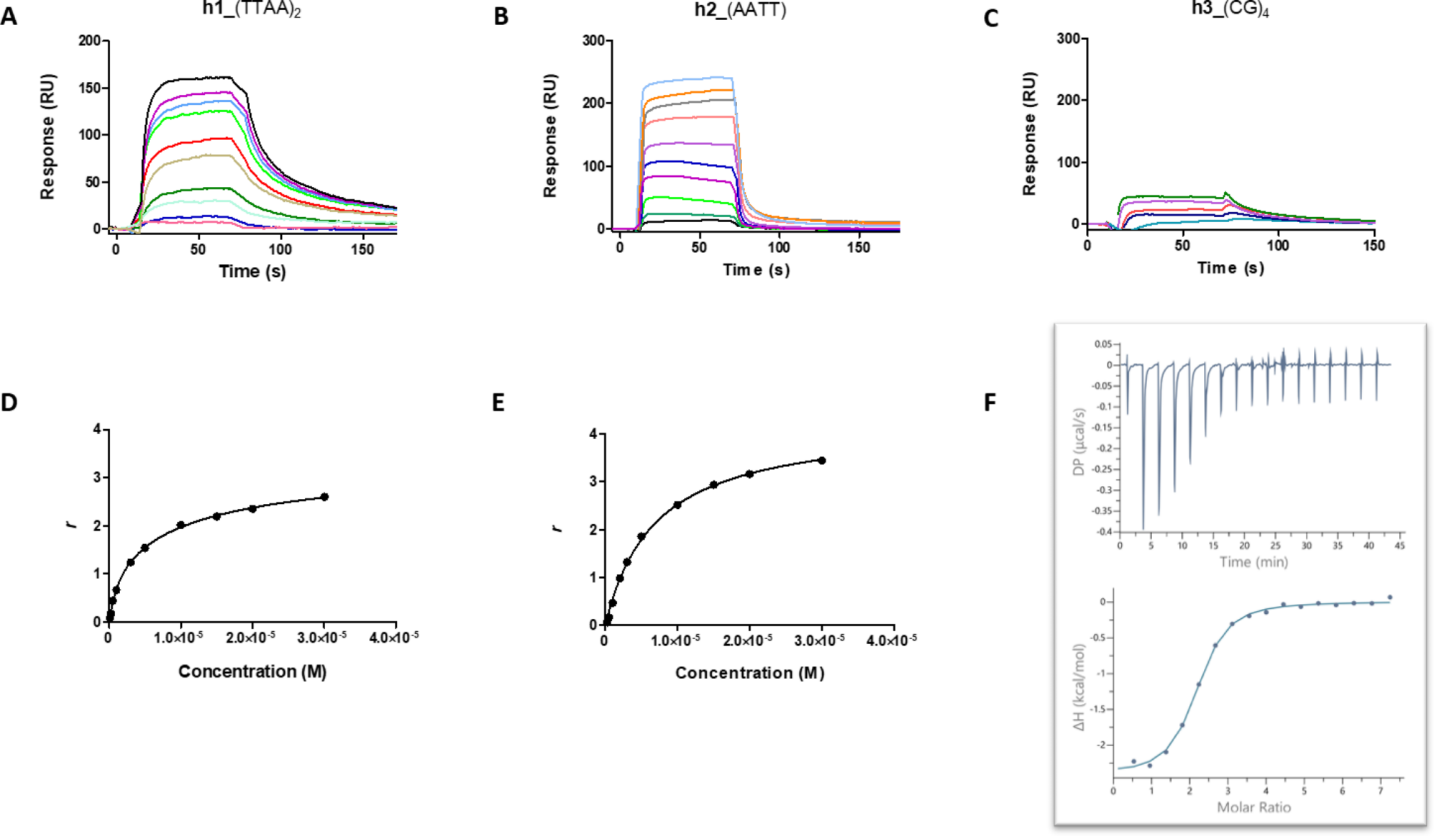
*Top panel*: SPR sensorgrams of AT-hook 1 (KRGRGRPRK) binding to (**A**) dsDNA 5’-biotin-CGTTAATTAACGC*CCCC*GTTAATTAACG [**h1**_(TTAA)_2_], (**B**) 5’-biotin-CGAATTCG*TCTC*CGAATTCG-3’ [**h2**_AATT], and (**C**) 5’-biotin-CGCGCGCG*TTTT*CGCGCGCG-3’ [**h3**_(CG)_4_] in MES + P20 (10 mM 2-(N-morpholino)ethanesulfonic acid), 1 mM EDTA, 100 mM NaCl, 0.005% surfactant P20, pH 6.25) at 25 ºC (the hairpin loop is italicised). *Lower panel*: SPR binding plots of AT-hook 1 with **h1**_(TTAA)_2_ (**D**) and **h2**_(AATT) (**E**) with fitting curve for a two-site affinity and one-site affinity models, respectively. The SPR response (RU) at equilibrium was converted to *r* (moles of bound compound per mole of DNA hairpin duplex; *r* = RU/RU_max_) and plotted against the free compound concentration, C_f_, flowing on the chip surface. Concentrations ranged from 0.05 to 30 µM for **h1**_(TTAA)_2_, 0.025 to 30 µM for **h2**_(AATT), and 0.5 to 10 µM for **h3**_(CG)_4_. (**F**) ITC experimental curves at 25 °C for titration of 0.9 mM AT-hook 1 into 30 µM d(5’-biotin-CGTTAATTAACG)_2_ duplex [**d2**_(TTAA)_2_] in MES buffer (MES 10 mM, EDTA 1 mM, NaCl 100 mM, pH = 6.25; T = 25 ºC). Top: raw ITC data (with buffer subtracted). Bottom: binding isotherm showing the dependence of successive enthalpy change per molar ratio of titrant (dots = experimental; line = fitting).

*AT-hook 1 binds preferentially to DNA at AT-rich sites.* The binding affinities of AT-hook 1 to dsDNA hairpins containing CGTTAATTAACG [**h1**_(TTAA)_2_], CGAATTCG [**h2**_AATT], and CGCGCGCG [**h3**_(CG)_4_] sequences (Fig. 1B) were determined by SPR-biosensor experiments. dsDNA hairpins were used because they are much more stable than DNA duplexes in the conditions of the assay where harsh surface regeneration conditions are used^24^. Each oligomer hairpin containing a 5’-covalently attached biotin group was immobilized on one channel of a streptavidin-coated sensor chip through biotin capture^8^. Solutions with known concentrations of AT-hook 1 were flowed on the chip surface until a steady-state was reached. The obtained sensorgrams for binding to **h1**_(TTAA)_2_ showed moderately fast kinetics of binding and slow dissociation (Fig. 3A). In contrast, binding and dissociation from the **h2**_(AATT) oligonucleotide was very fast (Fig. 3B). The AT-hook 1 peptide bound to **h1**_(TTAA)_2_ with a primary binding constant (*K*_1_, high affinity site) in the low micromolar range (*K*_1_ = 1.51 µM) and an approximately 9-times weaker secondary binding constant (*K*_2_/*K*_1_ = 9.5) (Table 2). In contrast, the binding of AT-hook 1 to **h2**_(AATT) was best fitted to a one-site model (*K* = 5.78 µM). This is compatible with the structure of this hairpin, which contains only one specific AATT site.

The observed binding of AT-hook 1 to the **h3**_(CG)_4_ sequence, which occurred at much higher concentration (i.e., no *K*_D_ could be calculated) compared with **h1**_(TTAA)_2_ and **h2**_AATT confirmed AT-selectivity and the capacity of AT-hook 1 to bind wider CG-containing grooves (Fig. 3C). Thus, the stoichiometry of binding to **h1**_(TTAA)_2_ and **h2**_(AATT) (*r* > 2 molecules per DNA duplex) that was observed in the SPR binding plots (Fig. 3D and E) may account for binding to the CG-rich hairpin loop, although nonspecific ionic interactions with DNA phosphate groups (as shown in the crystal structure) or binding to the major groove of the DNA hairpin^25^ cannot be excluded based on these SPR experiments (see below). However, it should be noted that crystallization experiments performed at high ligand concentration (up to 8:1) did not reveal interactions with the major groove of AT-rich DNA (unpublished data).

**Table 2:** DNA binding constants determined by SPR and ITC, and thermodynamic parameters determined by ITC at 25 °C for dsDNA^‡^ containing TTAATTAA, AATT, and (CG)_4_,

	SPR			ITC					
Compound	*K*_*d*_ (×10^-6^M)^*a, b*^				*K*_d_ (×10^-6^M)^*c*^	n (sites)	Thermodynamic parameters (kcal/mol)^*d*^		
	dsDNA **h1**_(TTAA)_2_	dsDNA **h2**_AATT	dsDNA **h3**_(CG)_4_	Oligo.			∆*H*	*-T*∆*S*	∆*G*
AT-hook 1 (KRGRGRPRK)	*K*_1_ = 1.51 *K*_2_ = 14.4	*K* = 5.78^*e*^	>10	Duplex **d2**	2.11 ± 0.29	2.06	− 2.43 ± 0.05	5.32	− 7.74
				Hairpin **h1**	3.15 ± 0.63	1.12	− 6.20 ± 0.29	− 1.30	− 7.51
**1**	*K*_1_ = 0.033 *K*_2_ = 108	*K*_1_ = 0.191 *K*_2_ = 985	>100^*f*^	Duplex **d2**	0.41 ± 0.08	0.68	− 6.51 ± 0.15	− 2.21	− 8.72
				Hairpin **h1**	1.05 ± 0.30	2.26	− 2.05 ± 0.07	− 6.11	− 8.16
**2**	*K*_1_ = 2.07 *K*_2_ = 421	*K*_1_ = 1.58 *K*_2_ = 113	>100^*f*^	Duplex **d2**	1.27 ± 0.34	0.69	− 5.54 ± 0.29	− 2.51	− 8.04
**3**	*K*_1_ = 0.23 *K*_2_ = 106	*K*_1_ = 0.42 *K*_2_ = 8660	>100^*f*^	Duplex **d2**	0.51 ± 0.11	0.68	− 7.39 ± 0.21	− 1.19	− 8.59
Pentamidine	*K*_1_ = 0.68 *K*_2_ = 38.8	nd^*g*^	nd		nd				

^‡^dsDNA hairpins used in the study (the loop is italicised): 5’-biotin-CGTTAATTAACG*CCCC*CGTTAATTAACG [**h1**_(TTAA)_2_], 5’-biotin-CGAATTCG*TCTC*CGAATTCG-3’ [**h2**_AATT], 5’-biotin-CGCGCGCG*TTTT*CGCGCGCG-3’ [**h3**_(CG)_4_]. ^*a*^Binding constants (*K*_1_) for fitting to a two-site binding model. ^*b*^The binding affinities are an average of two independent experiments and the experimental error is approximately ± 10%. The strong binding of **1** increases the error to approximately ± 50% (*n* = 3). ^*c*^*K*_d_ is the binding constant for the interaction of compounds **1**–**3** and AT-hook 1 with DNA **d2**_(TTAA)_2_ in MES buffer (pH = 6.25) at 25 ºC. The *K*_d_ values (duplicates) have an experimental error of approximately ± 10%. ^*d*^∆*G* (kcal mol^− 1^) is the binding free energy calculated from the equation ∆*G = -*RTlnK. *-T*∆*S* was calculated from ∆*G* = ∆*H* -T∆*S*. ^*e*^ Binding constants for fitting to a one-site binding model (*n* = 1). ^*f*^ Reference^8^. ^*g*^Not determined.

ITC was used to characterize the thermodynamics of binding of AT-hook 1 to DNA (Fig. 3F; Table 2). ITC assays provide the DNA-binding enthalpy (∆*H*) and allow the calculation of the free energy of binding (from Δ*G* = -RTlnK) and of the entropic (∆*S*) contribution to the binding Gibbs energy (from ∆*G* = ∆*H-*T∆*S*). As shown in Table 2, binding of AT-hook 1 to **d2**_(TTAA)_2_ has a low enthalpy contribution and it is mainly driven by entropy (T∆S = 5.32 kcal/mol) as was shown previously for AT-hook 1 binding to 5′-GGATATTGC*CCCC*GCAATATCC-3′^25^. This is expected for this kind of protein that barely shows secondary structure in solution before interaction with other macromolecules. The 2:1 AT-hook 1:DNA stoichiometry measured by ITC (Table 2) was consistent with SPR data (i.e., *r* ≥ 2 molecules per DNA duplex) and different from the X-ray structure which shows 1:1 stoichiometry in the space group P32. The structure of this **d2**_(TTAA)_2_ duplex, which lacks the C-containing loop found in the hairpin **h1**_(TTAA)_2_, rules out a mode of binding via these bases. Hence, the observed stoichiometry is consistent with a dual mode of binding via a specific AT-containing high affinity site (*K*_d_ in the range 1.51 [SPR] – 2.1 µM [ITC]) and a weaker less specific binding site that may account for ionic interactions with DNA phosphate groups (consistent with the X-ray structure). Contrary to Garabedian et al.^25^, we were unable to distinguish two binding sites in the ITC experiments, which is possibly due to relatively close binding constants as shown in the SPR assays (Table 2) and/or to the different DNA sequences used in Garabedian’s experiments (i.e., ATATT-containing hairpins). In fact, these authors acknowledged “significant uncertainty to the estimate of the binding affinities” they report in their work based on small values of binding enthalpy (ΔH ≈ 1–2 kcal/mol).^25^

Surprisingly, the thermodynamics and stoichiometry of binding to hairpin **h1**_(TTAA)_2_ were quite different to those observed with the analogue duplex **d2**_(TTAA)_2_ (Table 2, Figure S1B). In this case, AT-hook 1 formed a 1:1 complex with the DNA hairpin, the formation of which was mainly driven by enthalpy with a low entropy contribution (T∆S = -1.3 kcal/mol). This shows that the presence of the GC-loop in the **h1**_(TTAA)_2_ hairpin has profound influence on the binding mode of AT-hook 1. In earlier studies, Cui and Leng reported that HMGA2, which binds to AT-rich sites as a monomer under physiologically relevant salt conditions^26^, specifically recognizes a type of 15 bp AT-rich DNA sequence containing five AT-rich base pairs at the beginning, four GC-rich base pairs in the middle, and six AT-rich base pairs at the end of the consensus sequence^26^. They showed that the presence of the three segments in the consensus sequence were required for high-affinity binding.

### Characterization of compounds **1**–**3** binding and AT-hook 1 inhibition

**Fig. 4:**
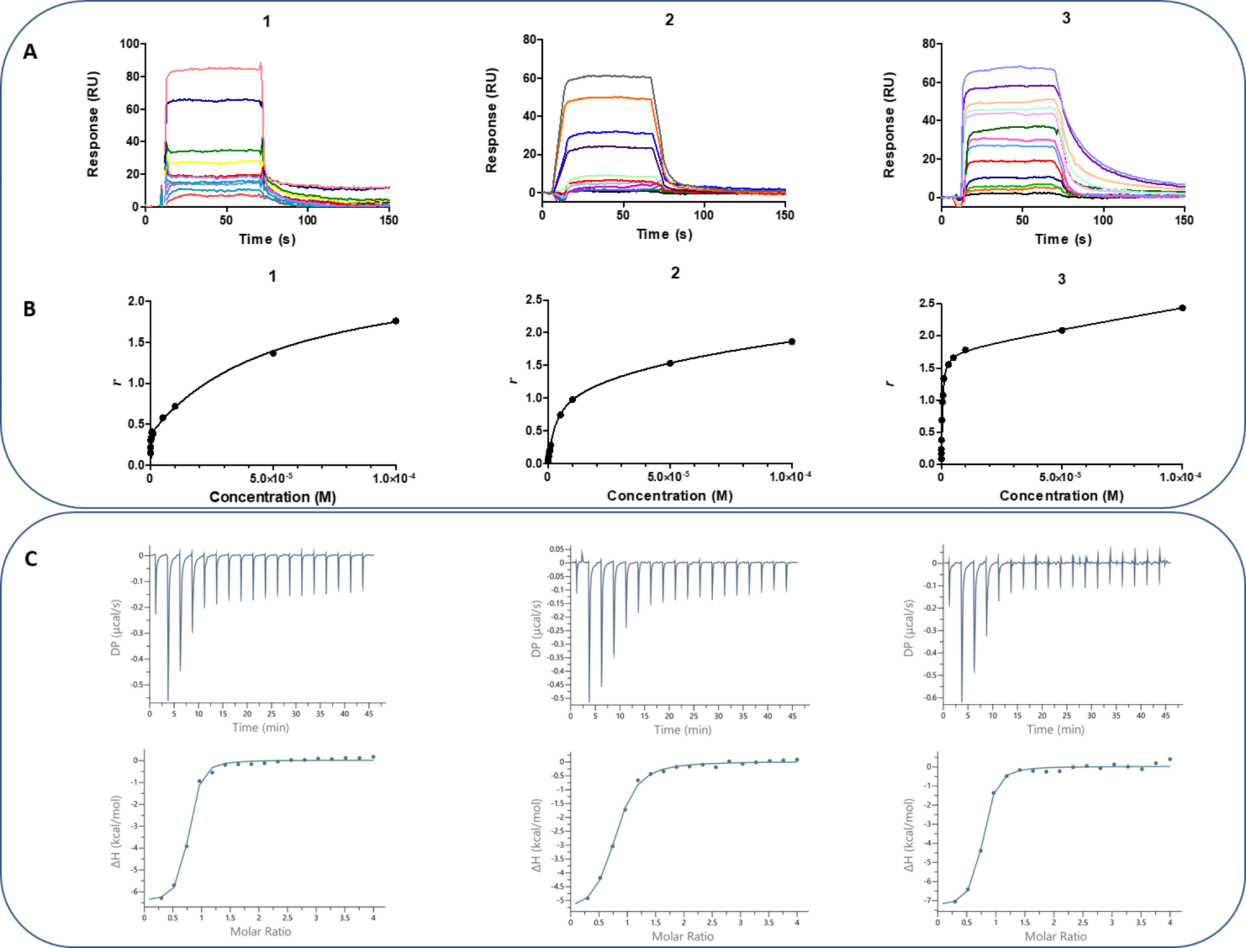
*Upper panel*: (**A**) SPR sensorgrams of compounds **1**–**3** binding to dsDNA hairpins biotin-CGTTAATTAACG*CCCC*CGTTAATTAACG [**h1**_(TTAA)_2_] in MES + P20 at 25°C (the hairpin loop is italicised). *Middle panel*: (**B**) SPR binding plots of compounds **1**–**3** with target DNA and fitting curve for a two-site affinity model. The SPR response (RU) at equilibrium was converted to *r* (moles of bound compound per mole of DNA hairpin duplex; *r* = RU/RU_max_) and plotted against the free compound concentration, C_f_, flowing on the chip surface. Concentrations ranged from 0.05 to 100 µM. *Lower panel*: (**C**) ITC experimental curves at 25°C for titration of 0.9 mM **1**, **2**, and **3** (from left to right) into 30 µM d(5’-biotin-CGTTAATTAACG)_2_ duplex [**d2**_(TTAA)_2_] in MES buffer (MES 10 mM, EDTA 1 mM, NaCl 100 mM, pH = 6.25; T = 25 ºC). Top: raw ITC data (with buffer subtracted). Bottom: binding isotherm showing the dependence of successive enthalpy change per molar ratio of titrant (dots = experimental; line = fitting).

*Compounds* ***1*** *–* ***3*** *bind preferentially to DNA at AT-rich sites.* The binding affinities of compounds **1**–**3** to dsDNA hairpins **h1**, **h2** and **h3** determined by SPR showed a similar binding behaviour (i.e., fast kinetics of binding and dissociation) (Fig. 4A). However, compound **3** showed slower dissociation phase from **h1**_(TTAA)_2_ on sensorgrams at high ligand concentration. This may indicate that part of the secondary binding energetics induces a conformational change in ligand structure that results in a more favourable minor groove complex with a slower k_off_^27^.

Compounds **1** and **3** bind selectively to AT- versus CG-containing DNA with primary binding constants (*K*_1_) in the submicromolar range and > 400-times weaker (*K*_2_/*K*_1_ > 400) secondary binding constants (Table 2; Fig. 4B). They bind **h1**_(TTAA)_2_ stronger than the control drug pentamidine (*K*_d_ = 0.68 µM) (Table 2). In contrast, **2** binds to **h1**_(TTAA)_2_ with a single digit micromolar affinity. Compound **1** binds to **h1**_(TTAA)_2_ more strongly (63 and 7-fold, respectively) than **2** and **3**. Interestingly, the binding affinity of **1** for this oligonucleotide was approximately 6-times stronger than for **h2**_AATT^28,29^.

Of note, the stoichiometry of binding (*r*) to the primary (high affinity) binding site was one molecule per DNA hairpin for the three compounds (Fig. 4B). At higher concentrations, the stoichiometry increased to > 1 ligand molecule per DNA duplex, which most probably accounts for unspecific binding to the hairpin loop^27^. The observed 1:1 stoichiometry in the ITC experiments with the TTAATTAA homodimer lacking the hairpin loop [i.e., **d2**_(TTAA)_2_] is consistent with this observation (Table 2). This hypothesis was confirmed in another ITC experiment where compound **1** was shown to form a 2:1 complex with the hairpin **h1**_(TTAA)_2_ (Table 2, Figure S1A).

The binding constants obtained by ITC, which were consistent with the primary binding constant values measured by SPR, confirmed the following binding strength order: **1** > **3** > **2** (Table 2). Titrations of compounds **1**–**3** into a **d2**_(TTAA)_2_ DNA solution provided plots of heat versus molar ratio which showed that the DNA duplex is completely saturated at 1:1 stoichiometry (Fig. 4C; Table 2). These results showed that both enthalpic and entropic contributions to the binding Gibbs energy are favourable for compounds **1**–**3**, indicating that they interact with the minor groove of DNA on the basis of a similar set of binding interactions. However, differences exist between the bis(2-aminoimidazoline) (**1**, **2**) and bis(arylimidamide) (**3**) compounds as shown by the thermodynamic data with **d2**_(TTAA)_2_ (Table 2). Among the bis(2-aminoimidazoline) derivatives, the relatively unfavourable ΔG for binding of **2** at 25 ºC is due to a less favourable binding enthalpy despite its slightly more favourable TΔS. In contrast, the more favourable binding of **1** comes from a combination of enthalpy and entropy terms. On the contrary, the binding of bisarylimidamide derivative **3** is clearly driven by a significantly more favourable (negative) ΔH whereas the low value of TΔS for complex formation (compared with **1** and **2**) reflects a weak loss in conformational entropy upon binding to DNA. Of note, the thermodynamics of binding of compound **1** to the hairpin **h1**_(TTAA)_2_ were opposite (i.e., low enthalpy and high entropy contribution) to that observed with the duplex **d2**. These findings illustrate the structural effect that the unpaired GC bases from the hairpin loop has on the DNA structure.

**Fig. 5:**
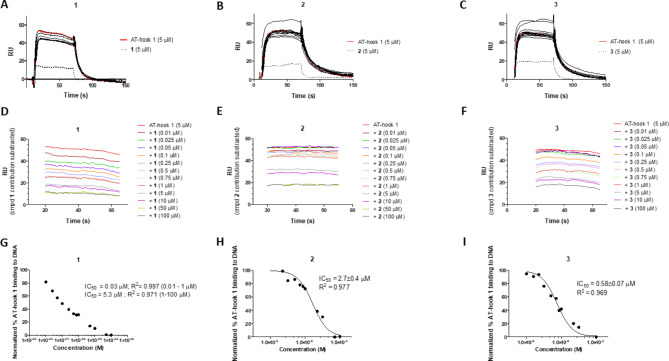
Competition experiments. *Upper panel*: SPR competition sensorgrams showing the inhibition of a fixed concentration (5 µM) of AT-hook 1 binding to dsDNA oligonucleotide **h1**_(TTAA)_2_ in the presence of increasing concentration of **1** (**A**), **2** (**B**), and **3** (**C**). Concentrations range from 0.01 to 100 µM. *Middle panel*: a decrease in RU signal with increasing concentration of compounds **1** (**D**), **2** (**E**), and **3** (**F**) is observed after subtraction of the compound’s contribution to the total SPR response. *Lower panel*: Inhibition curves and IC_50_ values for inhibition of AT-hook 1 binding to **h1**_(TTAA)_2_ by compound **1** (**G**), **2** (**H**), and **3** (**I**).

*Compounds* ***1*** *–* ***3*** *inhibit the binding of AT-hook 1 to AT-rich DNA.* To check if compounds **1**–**3** were able to displace the AT-hook peptide from its DNA binding site, we performed competition assays injecting mixtures of fixed concentration of AT-hook 1 (5 µM) and increasing concentrations of the tested compounds over the immobilised **h1**_(TTAA)_2_ DNA surface of the SPR sensorchip (Fig. 5A–C)^12,30^. The sensorgrams showed a decrease in RU signal (≈ 8.5 RU) when increasing the concentration of compound **1** at concentrations up to 1 µM, indicating a dose-dependent inhibition of AT-hook 1 binding to DNA (Fig. 5A). At higher concentrations, a dose-dependent increase in RU was observed due to the contribution of compound **1** to the bound mass on the sensor surface. A similar behaviour was observed with compounds **2** and **3** (Fig. 5B and C). By subtracting the compound’s contribution to the total RU signal at each concentration, a clear decrease in RU was observed with increasing concentration of compounds **1**, **2**, and **3** (Fig. 5D–F, respectively). This indicates that compounds **1**–**3** can displace AT-hook 1 from its DNA binding site.

To determine the IC_50_ values, the binding response in the steady-state region at each concentration was averaged and normalized as follows: 100% is the response (RU) with AT-hook 1 alone, and 0% is the response (RU) with saturation by the inhibitor. The IC_50_ values were determined by fitting the inhibition data with a model according to a competition system with 1:1 binding stoichiometry for AT-hook 1 and two site-binding for the competitor^30^. Compounds **1**, **2** and **3** inhibit AT-hook 1 binding to DNA with IC_50_ values of 0.03, 2.7 and 0.58 µM, respectively (Fig. 5G–I).

### Modelling studies: binding mode of compounds **1**–**3**

*Compounds***1***–* ***3*** *and AT-hook 1 bind in the same region of the* ***d1*** *_(TTAA)*_*2*_ *duplex*. The molecular docking studies of compounds **1**–**3** bound to **d1**_(TTAA)_2_ support a mode of binding where the molecules lie positioned in the upper part of the duplex and the central amide NH of the molecules points towards the floor of the minor groove. The compounds interact directly via hydrogen bonding to the bases and phosphate groups which line the surface of the minor groove, and also show van der Waals contacts with the floor and walls of the groove (Table S1, Figure S8). This predicted binding mode is similar to what was reported before for the complex of **1** with d[AAATTT]_2_^12^. Interestingly, compounds **1**–**3** bind in the same AT region (A10-T8) of the EF oligonucleotide duplex as Lys23 and Arg24 of AT-hook 1 (peptide G in the crystal structure). These findings are consistent with the observed binding inhibition in the competition experiments with AT-hook 1.

**Fig. 6:**
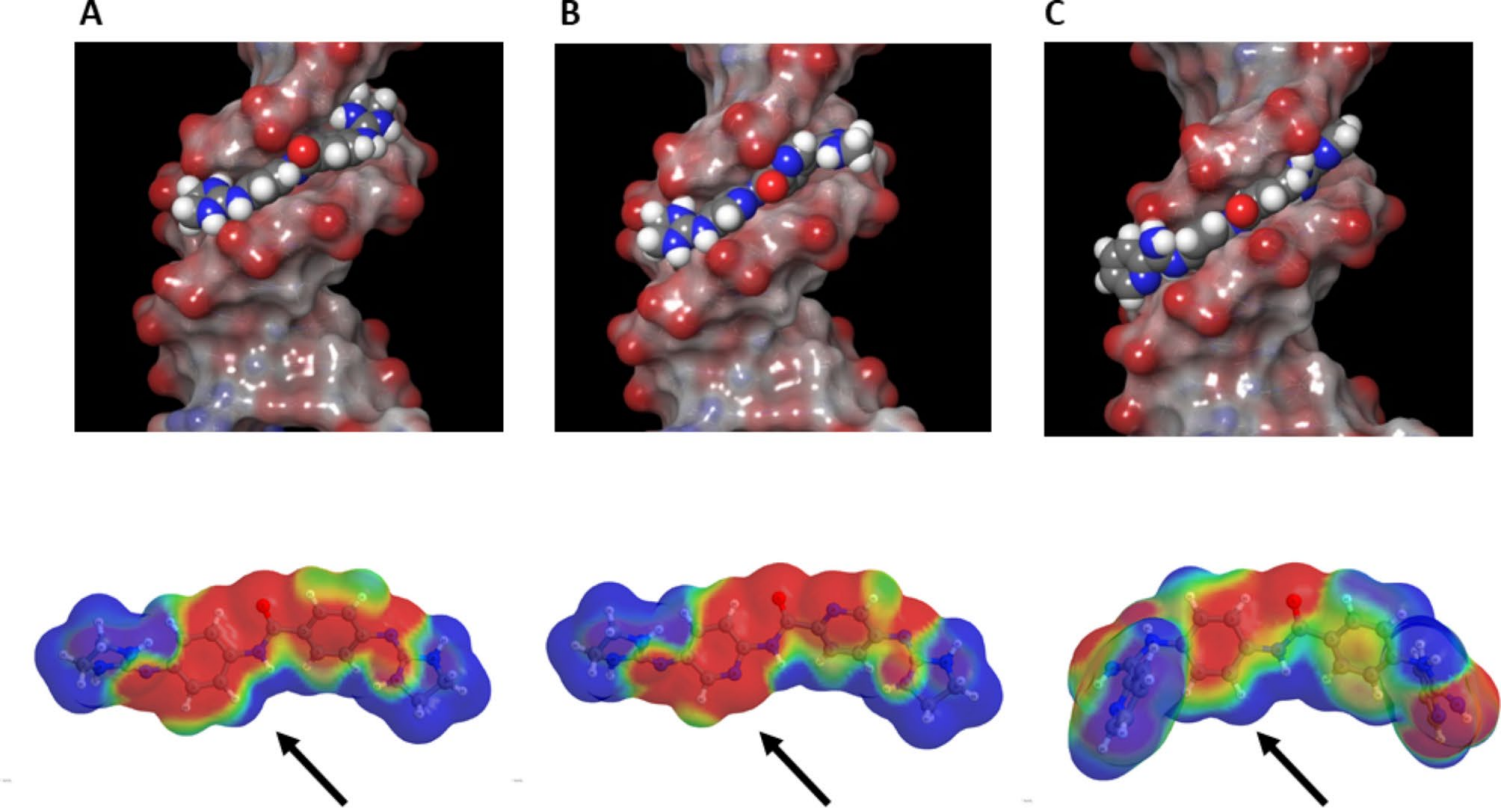
Upper panel: space filling model views of the docking poses of compound **1** (**A**), **2** (**B**), and **3** (**C**) bound to 8CPG. Lower panel: molecular electrostatic potential (MEP) surfaces of **1**, **2**, and **3** (from left to right) calculated with a B3LYP/6-31G(d) basis set (blue = positive, red = negative). The arrows indicate the main difference in MEP for the region of the molecule that interacts with the floor of the minor groove.

Despite of a similar orientation of the diphenylbenzamide scaffold within the minor groove, molecular docking predicts different hydrogen bonding patterns for the three molecules. Imidazoline derivatives **1** and **2** share a similar binding mode, being positioned between Adenine 10 (chain F) and Adenine 9 (chain E) of complementary chains. For compound **1**, one of the terminal cationic groups (NH6’) form hydrogen bonds with N3 of the adenine base A10 (**1**) whereas the other cationic group (NH5 and NH6) interacts with the phosphate backbone of thymine T8 (Supporting information: Figure S8 and Table S1). In contrast, the pyridine analogue **2** does not show hydrogen bond (HB) with the bases at the floor of the groove although one imidazoline cation forms HBs with the phosphate groups of A10 and A9. Remarkably, the introduction of N atoms (pyridine rings) affects the interaction with the floor of the minor groove as evidenced by the lack of H-bond interaction between NH(6’) and N3 of adenine A10. The presence of the pyridine N atoms also affects the geometry of the central scaffold as shown by the nearly coplanarity of the pyridine rings in **2** (178.3º) whereas the phenyl-containing compounds **1** and **3** have a slight torsion (170.9º and 169.1º, respectively, Table S1). These observations are consistent with the ITC results which showed that the relatively unfavourable ΔG for binding of **2** is due to a less favourable binding enthalpy (compared with **1** and **3**) despite its slightly more favourable entropic contribution. As regards to the bisarylimidamide compound **3**, one of the pyridine rings is aligned parallel to the walls of the minor groove with the amidine NH_2_ group pointing inwards. This arylimidamide group has close van der Waals interactions with sugar and phosphate atoms of the DNA backbones and the amidine NH_2_ makes two HBs with N3 of adenines A5 and A6 of the same strand (chain E). In contrast, the second pyridine ring, which is perpendicular to the walls of the groove and close to the groove mouth, shows a strong HB interaction with the phosphate backbone of A6 in the opposite strand (Figure S8). This twisted orientation of the terminal pyridine ring has been observed before with other bisarylimidamides (e.g., structure of the DB1880–DNA complex)^31^.

Ab initio calculations of the molecular electrostatic potential (MEP) helped rationalize the observed binding modes and measured affinities. Compounds **1** and **3** have a more electropositive MEP in the region of the molecule opposite to the amide carbonyl group. This results in more favourable interactions with the more electronegative floor of the minor groove. In contrast, the presence of the pyridine N atom in **2** results in unfavourable electronegative potential in the pyridine region facing the groove’s floor (Fig. 6).

## Antikinetoplastid activity

Bis(2-aminoimidazoline) compounds **1** and **2** display disparate antiprotozoal activities despite of similar structures. Compound **1** is 30-times more potent than **2** against *T. brucei* (EC_50_ = 0.83 and 25.5 µM, respectively) and it shows low micromolar activity against promastigotes of *L. donovani* whereas **2** is inactive against this parasite (Table S3). Remarkably, the AT-DNA binding affinity and AT-hook 1 inhibitory potency of **1** are approximately two orders of magnitude stronger than that of compound **2**. Thus, the presence of two pyridine rings in **2** has a detrimental effect on both DNA binding (*vide supra*) and biological activity. On one hand, both compounds are dicationic at physiological pH, even though **1** is slightly more basic (p*K*_a_ = 9.2/10.26) than **2** (p*K*_a_ = 8.08/9.24),^8^ which is favourable for accumulation in the mitochondrion of the parasite and binding to kDNA^32^. On the other hand, compound **1** is somewhat less polar (exp. logP = 0.21; Topological polar surface area (TPSA) = 101.9)^8^ than **2** (clogP = -0.91; TPSA = 126.7) which may enhance the hydrophobic interactions with the wall of the minor groove, resulting in stronger binding. Since the kinetoplast of *T. brucei* is a target of compound **1**^12^, these results suggest that the lower AT-DNA binding affinity of **2** is a contributing factor to its weaker antitrypanosomal activity.

As regards to the bis(arylimidamide) compound **3**, its superior antiprotozoal activity against the three kinetoplastid parasites (including low micromolar to submicromolar activities against intracellular forms of *T. cruzi* and *L. donovani* as shown in Table S3) cannot be attributed solely to its submicromolar DNA binding affinity and/or AT-hook protein inhibition. This compound has distinct physicochemical properties (p*K*_a_ = 6.61/7.68; logP = 2.76; TPSA = 126.6) and it will be partially charged at physiological pH^8^. Hence, other factors such as cell uptake differences and maybe different cellular targets must be considered. For instance, other studies implicated CYP5122A1, a cytochrome P450 associated with ergosterol metabolism in *Leishmania*, in the antileishmanial action of arylimidamides^33^.

## Discussion

In living cells, a dynamic network of proteins such as HMGs or histone H1 can bind to and remodel chromatin structure^30,34^. HMGA are abundant nonhistone architectural nuclear factors that are involved in the modulation of chromatin structure and gene expression, thus influencing a vast array of biological processes including growth, proliferation, differentiation and death^14,15^. On the other hand, their abnormal expression has been implicated in several diseases, including cancer. Thus, high levels of these proteins correlate with increased malignancy and metastatic potential in a wide range of cancer types, and the use of minor groove binders that can compete with HMGA for binding to AT-rich DNA has been proposed as an anticancer strategy^35–37^. In trypanosomatid parasites, different AT-hook (e.g., LamAT-Y) and HMG-box proteins (TbKAP6, TcHMGB) have been characterized and shown to be essential for parasite biology^13,20,38^.

The elucidation of the crystal structure and the characterisation of the binding strength and thermodynamics of formation of the AT-hook 1/DNA complex that is reported here shed light on the molecular basis of the interaction between AT-hook proteins and DNA. Importantly, the three AT-hook 1 peptides observed in the asymmetric crystal unit display distinct conformations: two of them (G, H) show the expected AT-hook conformation whereas the third one (I) does not interact with the grooves of the DNA duplexes and displays ionic interactions with DNA phosphate groups instead. This could indicate that AT-hook 1 has a double function recognizing AT regions and acting as crosslinking agent between different DNAs. This is different to what was observed in the crystal structure of AT-hook 3 bound to AT-DNA, where four peptides bound in an identical conformation to a bent and widened DNA molecule^19^. In the AT-hook 1/DNA complex, a minor groove widening (calculated with 3DNA)^39^ is observed in the region occupied by the AT-hook with P-P distances of 11.5–11.8 Å (vs. 12–13 Å for AT-hook 3/DNA complex^19^), whereas the DNA region not interacting with AT-hook 1 is narrow with P-P distances from 8.4 to 9.7 Å. The width of the minor groove depends on the DNA sequence and also on its interaction with proteins^40^. Arginine residues play an important role in the protein–DNA recognition process; if the arginine is fully placed inside the minor groove, the groove is generally narrower. In the case of AT-hook 3 interacting with CGAATTAATTCG, the arginine side chain is fully placed inside the minor groove^19^ which is widened to accommodate the guanidinium and methylene groups of the side chain (Figure S7). In contrast, the arginine residue of AT-hook 1 interacts with CGTTAATTAACG from outside, being the guanidinium cation the only group in contact with the thymine (T8) oxygen in the minor groove. As a consequence, the electrostatic repulsion among phosphates is reduced and the minor groove is narrower than in AT-hook 3. These results agree with the data reviewed by Rohs et al.^40^, showing that, in the case of AATT and TTAA sequences, the minor groove width of DNA has a high variability. These data suggests that HMGA is a versatile protein which different AT-hooks can act together to promote DNA compaction. These results are consistent with the recent findings on the role of HMGs as regulator of chromatin organization and function. For instance, the AT-hook 1 was shown to be essential for chromatin condensation by HMGA2^41^. Moreover, protein-DNA interactions were shown to promote phase separation of HMGA1a through electrostatic interactions via AT hooks 2 and 3^42^.

A comparison between the structures of AT-hook 1 and AT-hook 3 is shown in Figure S7. The overall conformation is rather similar with two significant differences; in particular, the conformation of the chain next to the central glycine is different. The X-ray structure of AT-hook-3 shows that the peptide enters more deeply into the minor groove and displays different orientation of the main chain NH groups, which form hydrogen bonds with thymines in the DNA. The presence of such hydrogen bonds was postulated in the work of Reeves and Nissen^17^. An additional difference is the position of the guanidinium group of Arg26, which is not uniquely positioned in the X-ray structure of AT-hook 1. However, the main lesson of these works is that the AT-hook can bind to a distorted DNA molecule.

The binding stoichiometry of AT-hook 1 with **d2**_(TTAA)_2_ in solution (i.e., SPR and ITC experiments) was > 2. AT-hook 1 binds to the minor groove of AT-DNA with high affinity whereas another molecule binds with lower affinity, possibly via ionic interactions with DNA phosphate groups. On the other hand, the differences observed by SPR in the kinetics of AT-hook 1 binding (and dissociation) to **h1**_(TTAA)_2_ and **h2**_AATT may perhaps indicate a sequential mode of binding to **h1**_(TTAA)_2_ which has an extended AT-region compared with **h2**. According to the crystal structure, AT-hook 1 interacts with the minor groove via the R2-G3 region which enters into the AT region of the duplex (Fig. 2D and S2-S6). In addition, the neighbouring K1 and R4 residues interact with the backbone phosphates and increase the stability. Hence, it is possible that a dynamic equilibrium takes place where one molecule of AT-hook 1 binds to one and two TTAA sites alternatively as shown in Figure S9.

The fundamental characteristic shared by small MGB molecules is the presence of an aromatic framework with a matching concave shape that can form hydrogen bonds and fits into the convex minor groove. At physiological pH, the protonated amines of diamidine or diguanidine ligands interact with the strong negative electrostatic potential of helical B DNA in the AT-rich regions. Van der Waals interactions stabilize the drug-DNA combination in addition to hydrogen bonds and electrostatic interactions^43,44^. The DNA minor groove binding compounds **1**–**3** have the same “flat” diphenyl (or dipyridyl) amide core but different heterocyclic cationic groups at both ends of the molecule. Under physiological conditions compounds **1**–**3** are mostly dicationic and display a classical type curvature for minor groove binding (136º, 140º, and 145º, respectively) as measured according to the protocol described by Wilson & Paul^44^. Consistent with the SPR, ITC and docking experiments, they bind to the minor groove of DNA on the basis of a similar set of interactions, although **2** and **3** are slightly less potent MGBs. The predicted binding mode for the bis(2-aminoimidazoline) derivatives **1** and **2** is similar to what was observed previously in the complex of **1** with d[AAATTT]_2_ in which the NH groups of the imidazoline rings form HBs with adenine N3 and thymine O2 of the DNA^12^. Such binding interactions were also reported for the 4,4’-bis(imidazolinylamino)diphenylamine analogue bound to d(AAAATTTT)_2_ or 5’-d(CTTAATTCGAATTAAG)^45,46^. Interestingly, compounds **1**–**3** display distinct sequence selectivity depending on the central core of the molecule. The importance of the scaffold of the molecule for sequence selectivity was observed previously with antitrypanosomal bisguanidines. In these series, the 1,3-diphenylurea^47^ or diphenylamine^46^ linker did not provide sequence selectivity contrary to the N-phenylbenzamide scaffold which showed higher affinity towards 5’-AATT over 5’-ATAT sequences. For bis(2-aminoimidazoline compounds, enhanced selectivity to AATT sites can also be obtained via N-hydroxylation of the imidazoline ring^28^. Compounds **1** and **3**, which possess the same N-phenylbenzamide scaffold and different cationic groups, bind (TTAA)_2_ more strongly than AATT. In contrast, compound **2**, which has the same 2-aminoimidazolinium cation as **1** and a dipyridyl amide scaffold, is 5-fold more selective towards the AATT sequence. The basis of this selectivity can be explained by the difference in minor groove width among these sequences. Since narrow minor grooves strongly increase the negative electrostatic potential of the DNA^40^, the interaction of **1** and **3** with (TTAA)_2_ is favoured with respect to the wider AATT minor groove (i.e., 9.3–10.5 Å, PDB: 1BNA)^48^ because these compounds display a more electropositive MEP in the region of the molecule interacting with the floor of the groove (Fig. 6). On the other hand, a stronger solvatation of **2** due to the presence of two extra pyridine N atoms may also contribute to the lower binding affinity of this compound in an aqueous environment. Similar trends have been observed previously with DB667 arylimidamide analogues binding to AT sequences of four to six base pairs^31^.

The capacity of compound **1** to displace the HMGA1a protein from its DNA binding site was reported previously (IC_50_ = 6.0 µM)^12^. In these competition experiments, a Δ50–91 version of the HMGA1a protein, which contained only the AT-hook 2 and 3 domains and no C-terminal acidic domain was used with a AATAAT_ATTATT-containing oligonucleotide with two distinct binding sites^12^. In the current SPR experiments, we showed that compound **1** inhibited AT-hook 1 binding to the high affinity AT-site of **h1**_(TTAA)_2_ with a nanomolar range IC_50_ value (30 nM). Therefore, this experiment confirms that **1** is a potent binding inhibitor of the three AT-hooks of HMGA1a. Interestingly, the inhibition curve showed an inflexion point (plateau) at a concentration near 1 µM. Hence, two IC_50_ values could be calculated (0.03 and 5.3 µM) which may correspond to the inhibition of AT-hook 1 binding to the high affinity (minor groove) and low affinity (hairpin loop) binding sites of the hairpin (*K*_D_ = 1.51 and 14.4 µM, respectively). Indeed, we showed that compounds **1**–**3** bind to the DNA hairpin **h1**_(TTAA)_2_ with a 2:1 ligand: DNA stoichiometry whereas compound **1** forms 1:1 complex with the linear duplex **d2**_(TTAA)_2_. These data are consistent with a specific minor groove mode of binding to the AT sequence and a nonspecific binding to the GC hairpin loop^27^.

From a medicinal chemistry point of view, the incorporation of pyridine rings in dicationic MGBs has shown its potential to improve the blood-brain barrier permeability of diamidines active against *T. brucei* and *Leishmania* parasites^49,50^. However, contrary to previously reported aza analogue diamidines, which were as potent in vitro as their diamidine counterparts^51^, the presence of pyridine rings in the structure of **2** is clearly detrimental for DNA binding and in vitro activity against trypanosomes and *Leishmania* parasites. These results show that the SAR results from apparently “similar” MGBs with different cationic groups (e.g., aza analogue diamidine vs. bis(2-aminoimidazoline)) cannot be extrapolated.

The targets involved in the antiparasitic action of other minor groove binding bis(arylimidamides) similar to **3** are still subject of debate. For instance, their anti-*T. cruzi* activity does not correlate with their binding affinity to kDNA and their action against *T. cruzi* intracellular amastigotes leads to disruption of kDNA but also vacuolization^52^. Daliry et al.^52^. have shown that some highly active bisarylimidamides (e.g. DB766) can deeply alter the parasite kDNA topology, *“suggesting that specific topological effects are more relevant to the biological activity of amidines than the simple binding affinity characteristics.”* We have shown earlier that bis(2-aminoimidazoline) compound **1** exerts its trypanocidal action by altering the integrity of *T. brucei* kDNA^12^. Since the three compounds studied here display potent antiparasitic activities against *T. brucei*, *T. cruzi*, and *L. donovani* and they can compete with AT-hook 1 binding to DNA, it is possible that AT-hook proteins (e.g., LamAT-Y) be involved in their mode of action against *Leishmania* parasites^20^. However, cell uptake differences and other cellular targets may also be involved to explain the different susceptibilities of *Leishmania* and *T. cruzi* parasites against both classes of compounds. Thus, further investigations on the mode of action of these compounds are warranted.

## Conclusions

The elucidation of the crystal structure of the first AT-hook 1 of the HMGA proteins complexed with DNA is an important step towards the understanding of the mode of action of these intrinsically disordered proteins of great biological relevance. The biophysical studies in solution showed that AT-hook 1 forms an entropy-driven 2:1 complex with (TTAA)_2_-containing DNA with relatively slow kinetics of dissociation. The propensity of AT-hook 1 to bind preferentially to the minor groove of AT-rich DNA was confirmed by SPR and ITC, even though its capacity to bind wider CG-containing sequences (less specifically) was also established.

We showed that small molecule DNA minor groove binders with antiparasitic activity can inhibit the interaction between AT-hook 1 and DNA. These data provide clues regarding a possible mode of action of these compounds through the inhibition of AT-hook proteins relevant in these parasites (e.g., LamAT-Y in *Leishmania* parasites). In fact, such inhibitory capacity is relevant not only for antiparasitic chemotherapy but also in the field of cancer because HMGA proteins are involved in neoplastic transformation and tumour progression. Among these structurally related compounds, the bis(2-aminoimidazoline) compound **1** is a stronger binder and better AT-hook 1 inhibitor than bis(arylimidamide) **3**, although this does not translate into superior antikinetoplastid activity. Hence, other factors such as cell uptake differences and/or different cellular targets must be considered to explain these discrepancies. In contrast, the lower AT-DNA binding affinity of **2**, which is related to the presence of two pyridine rings in the scaffold, could contribute to its weaker antitrypanosomal activity.

## Methods

### Crystallization assays

#### Oligonucleotide Synthesis

Peptide acetyl-KRGRGRPRK-amide was synthesized at the Organic Chemistry Dept. of Pompeu Fabra University, Barcelona. The deoxyoligonucleotide d(CGTTAATTAACG)_2_ [**d1**_(TTAA)_2_] was synthesised at the Pasteur Institute as the ammonium salt on an automatic synthesiser by the phosphoramidite method. It was purified by gel filtration and reverse-phase HPLC.

#### Crystallization

The crystal was grown by vapour diffusion at 4 ºC in a hanging-drop containing 0.2 mM DNA duplex, 0.8 mM peptide, 10 mM MgCl_2_, 0.025 mM NiCl_2_, 25 mM Na cacodylate buffer (pH 6.0) and 2.5% 2-methyl-2,4-pentanediol (MPD) equilibrated against a 17% MPD reservoir. MPD acts both as a precipitant and as a cryoprotectant. The MPD concentration of the reservoir was increased gradually up to 37.6%. The temperature was initially decreased from 20 ºC to 16 ºC, 11 ºC and finally left at 4 ºC. After 18 months, a single-crystal was flash-frozen and stored in liquid nitrogen until diffraction.

#### Data collection and structure determination

A PILATUS 6 M detector on beamline BL13-XALOC^53^ at the ALBA synchrotron, Barcelona, was used for data collection at 100 K and a wavelength of 0.979 Å, with a maximum resolution of 1.40 Å. The data were integrated using *Autoproc aniso* v1.0.5 and scaled with *Aimless Pointless*^54^ from the *CCP4* suite (v7.0.074)^55^. The space group turned out to be the P 32. A B-DNA model (PDB: ID 5M68) Watson-Crick base-pair pairing was used as starting search model for molecular replacement with *PHASER* (v 2.8.3)^56^. The replacement was next refined with *REFMAC5* (v5.8.0238)^57^ and real space refinement was performed with *Coot*^58^. The coordinates and stereochemical restraint dictionary of Peptide molecules were generated with *Phenix*^59^ and one peptide per duplex was placed in the minor groove of the DNA structure using *Coot.* Several cycles of maximum-likelihood isotropic restrained refinement were performed with *REFMAC5* (maximum resolution 1.40 Å). Finally, a last round of refinement with anisotropic restrictions was completed to obtain the final values of R _work_ = 0.124 and R _free_ = 0.158 in a resolution range of 37.79–1.40 Å with a completeness of 99.8%. Four magnesium ions were detected. Solution coordinates have been deposited in the Protein Data Bank as PDB-ID: **8CPG**. The DNA structural parameters were calculated using the *3 DNA* software^39^.

#### Surface plasmon resonance (SPR)–biosensor experiments and isothermal titration calorimetry (ITC) experiments

The 5’-biotin labelled DNA hairpins (**h1**, **h2**, and **h3**) were purchased from International DNA Technologies (IDT) with reverse-phase HPLC purification. The DNA hairpin sequences included 5’-biotin-CGTTAATTAACG*CCCC*CGTTAATTAACG [**h1**_(TTAA)_2_], 5’-biotin-CGAATTCG*TCTC*CGAATTCG-3’ [**h2**_AATT], and 5’-biotin-CGCGCGCG*TTTT*CGCGCGCG-3’ [**h3**_(CG)_4_]. The oligomers were dissolved in MES (10 mM 2-(N-morpholino)ethanesulfonic acid), 1 mM EDTA, 100 mM NaCl, pH 6.25. The AT-hook 1 nonapeptide (H-KRGRGRPRK-OH, > 98% purity) was purchased from ThermoFisher. Compounds **1**–**3** were synthesised previously^8,10^. AT-hook 1 and compounds **1**–**3** stock solutions were prepared in the MES buffer described above at 5 mM (AT-hook 1), 10 mM (**1** and **2**) and 2.5 mM (**3**).

#### SPR-biosensor experiments: binding affinity and binding inhibition

SPR binding and competitive experiments were performed at 25 °C with a Biacore X-100 apparatus (Biacore GE). Running buffer containing MES + P20, consisting of MES supplemented with surfactant P20 (10 mM 2-(N-morpholino)ethanesulfonic acid, 1 mM EDTA, 100 mM NaCl, 0.005% surfactant P20, pH 6.25), was used for the experiments with AT-hook 1 peptide and compounds **1**–**3**. The DNA hairpins were immobilised on a streptavidin-derivatised gold chip (SA chip from Biacore) by injection of a 25 nM hairpin DNA solution with a flow rate of 1 µL/min until approximately 400 RU were reached. Flow cell 1 was used as reference while flow cell 2 was immobilised with the hairpins in different chips. Direct binding of AT-hook 1 and compounds **1**–**3** was measured by injection of increasing concentrations over the immobilised DNA surfaces at a flow rate of 50 µL/min for a period of 60 s followed by a dissociation period of 120 s. Regeneration of the surface was made with NaCl 200 mM/NaOH 10 mM using a flow rate of 10 µL/min during 30 s. The RU values in the steady-state region of the sensorgrams at each concentration were averaged over a 10-s time zone and converted to *r* (moles of compound bound per mole of DNA hairpin). The binding affinity was determined by fitting the results to a two-sites binding model according to the equation:

$$r={\text{ }}({K_1}{C_f}\,+\,{\text{2}}{K_1}{K_2}{C^2}_{f})/({\text{1}}\,+\,{K_1}{C_f}\,+\,{K_1}{K_2}{C^2}_{f}),$$

where *r* is the moles of bound compound per mole of DNA hairpin duplex, *C*_*f*_ is the free concentration at the equilibrium, and *K*_1_ and *K*_2_ the microscopic binding constants.

The competition experiments were prepared with samples containing a fixed concentration of AT-hook 1 peptide (5 µM) and a series of concentrations of compound **1**–**3** ranging from 0.01 to 50 µM in MES buffer. The samples were injected to the immobilised DNA surfaces at a flow rate of 50 µL/min for a period of 60 s followed by a dissociation period of 150 s. The regeneration conditions were similar to the binding experiments described above. The corrected SPR response values (RU) for AT-hook 1 binding at steady state were averaged over a 10-s time zone and normalised by setting the RU with the AT-hook 1 peptide alone as 100% binding to the specific binding site, and the RU with saturation by the inhibitor as 0% AT-hook 1 specific binding to DNA^30^.

IC_50_ values were determined by fitting the inhibition data with a model according to a competition system with 1:1 binding stoichiometry for AT-hook 1 peptide and two site-binding for competitor:

$$\% {\text{ peptide binding to DNA}}\, = \,{\text{1}}00/[{\text{1}}\, + \,{\text{C}}({\text{1}}\, + \,{\text{K}}_{{{\text{c2}}}} {\text{C}})/[{\text{IC}}_{{{\text{5}}0}} ({\text{1}}\, + \,{\text{K}}_{{{\text{c2}}}} {\text{IC}}_{{{\text{5}}0}} )]]$$

where K_c2_ is a macroscopic binding constant for inhibitor binding to DNA, IC_50_ is the concentration of the inhibitor that causes 50% inhibition of AT-hook 1 binding to DNA, and C is the concentration of inhibitor^12^.

#### Isothermal titration calorimetry (ITC)

The oligonucleotide 5’-biotin-CGTTAATTAACG-3’ was purchased from International DNA Technologies (IDT) with reverse-phase HPLC purification. The self-complementary duplex [**d2**_((TTAA)_2_] was annealed as follows: a solution of the oligonucleotide dissolved in MES buffer (10 mM 2-(N-morpholino)ethanesulfonic acid, 1 mM EDTA, 100 mM NaCl, pH 6.25) was heated at 94 ºC (water bath) for 2 min, removed from heat and allowed to cool to room temperature.

Isothermal titration calorimetry (ITC) experiments were performed on a PEAQ-ITC instrument. In each experiment 2 µL injections of 0.9–1.2 mM AT-hook 1 nonapeptide or compounds **1**–**3** were made to a 200 µL sample cell containing 30 µM of DNA duplex **d2**_(TTAA)_2_ or hairpin **h1**_(TTAA)_2_ in MES buffer (10 mM 2-(*N*-morpholino)ethanesulfonic acid, 1 mM EDTA, 100 mM NaCl, pH 6.25). Experiments were performed at 25 °C with injections at 300-s intervals and 750 rpm stirring speed. Control experiments (injecting ligand solutions into the buffer) were conducted to obtain a baseline for each experiment and determine the heat of dilution/mixing. These values were subtracted from the experimental runs in the presence of protein. Analysis of the data was performed using PEAQ-ITC analysis program. Titrations were performed using at least two different preparations (*n* ≥ 2).

#### Ab initio calculations

Optimizations were carried out with the M06-31G(d) DFT functional^60^ basis set^61^ using the scientific software Gaussian16^62^. Frequency calculations of all optimized systems at the same level of theory confirm that they were minima (no imaginary frequencies) or TSs (only one imaginary frequency). The Molecular Electrostatic Potential (MEP) was computed on the 0.001 a.u. electron density isosurface with the Multiwfn^63^ software, using the B3LYP/6-31G* wavefunction.

## Electronic Supplementary Material

Below is the link to the electronic supplementary material.

Supplementary Material 1

## Data Availability

The datasets generated during and/or analysed during the current study are available from the corresponding author on reasonable request.

## Abbreviations

EDTA: Ethylenediaminetetraacetic acid
HB: Hydrogen bond
HMG proteins: High mobility group proteins
ITC: Isothermal titration calorimetry
kDNA: Kinetoplast DNA
MEP: Molecular electrostatic potential
MES: 2-(N-morpholino)ethanesulfonic acid
MGB: Minor groove binder
MPD: 2-methyl-2,4-pentanediol
SAR: Structure-activity relationships
SPR: Surface plasmon resonance
TPSA: Topological polar surface area
