# Supplementary material for: Crystal structure of the HMGA AT-hook 1 domain bound to the minor groove of AT-rich DNA and inhibition by antikinetoplastid drugs
Source: Sci Rep. 2024 Oct 30;14:26173. doi: 10.1038/s41598-024-77522-3 (PMC11526092; doi:10.1038/s41598-024-77522-3)
Supplement: Supplementary file 1 — Supplementary Material 1 [file 41598_2024_77522_MOESM1_ESM.pdf]

Electronic Supporting Information

Crystal structure of the HMGA AT-hook 1 domain bound to the minor groove of AT-rich DNA and inhibition by antikinetoplastid drugs

J. Jonathan Nué-Martinez[1,‡], Marta Maturana[2,‡], Laura Lagartera[1,‡], Juan-Antonio Rodríguez Gutiérrez[1], Roeland Boer[3], J. Lourdes Campos[2], Núria Saperas[2,*] and Christophe Dardonville[1,*]

Table of contents:

- Figure S1: ITC experimental curves for titration of **1** and AT-hook 1 into hairpin duplex [**h1_**(TTAA)$_2$]

- Details of the peptide-DNA interactions in the crystal (PDB: 8CPG): Figures S2–S7

- Molecular docking studies: experimental part, Figure S8, and Tables S1–S2.

- Table S3: activity of compounds **1**–**3** against *T. brucei*, *T. cruzi* and *L. donovani*.

- Figure S9: putative binding model of AT-hook 1 with (TTAA)$_2$ duplex in solution.

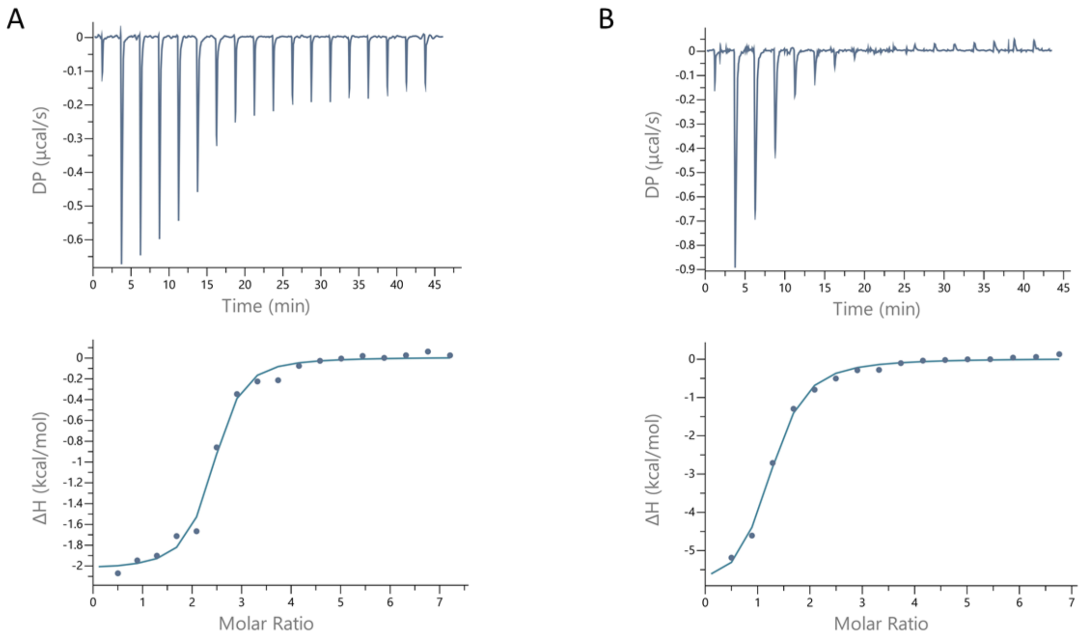

Figure S1. ITC experimental curves at 25 ºC for titration of 0.9 mM **1** (A) and AT-hook 1 (B) into 30 µM hairpin duplex [**h1**_(TTAA)$_2$] in MES buffer (MES 10 mM, EDTA 1 mM, NaCl 100 mM, pH = 6.25; T = 25 ºC). Top: raw ITC data (with buffer subtracted). Bottom: binding isotherm showing the dependence of successive enthalpy change per molar ratio of titrant (dots = experimental; line = fitting).

Details of the peptide-DNA interactions in the crystal (8CPG)

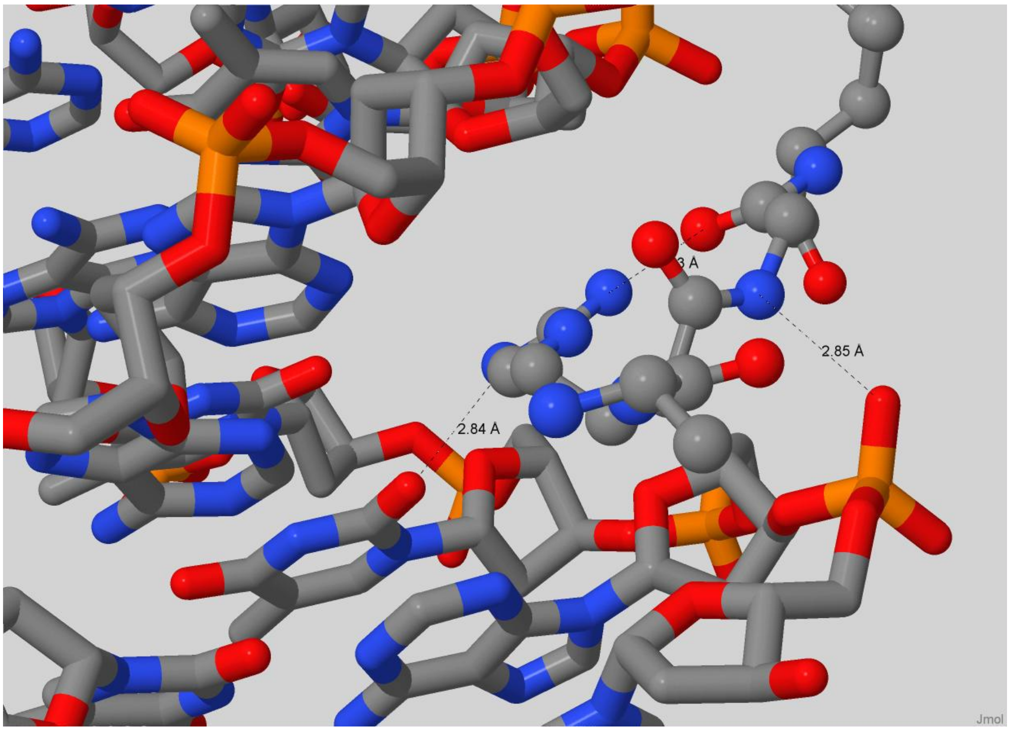

Figure S2. Hydrogen bond between the guanidinium group of Arginine 24 and the O2 oxygen of thymine.

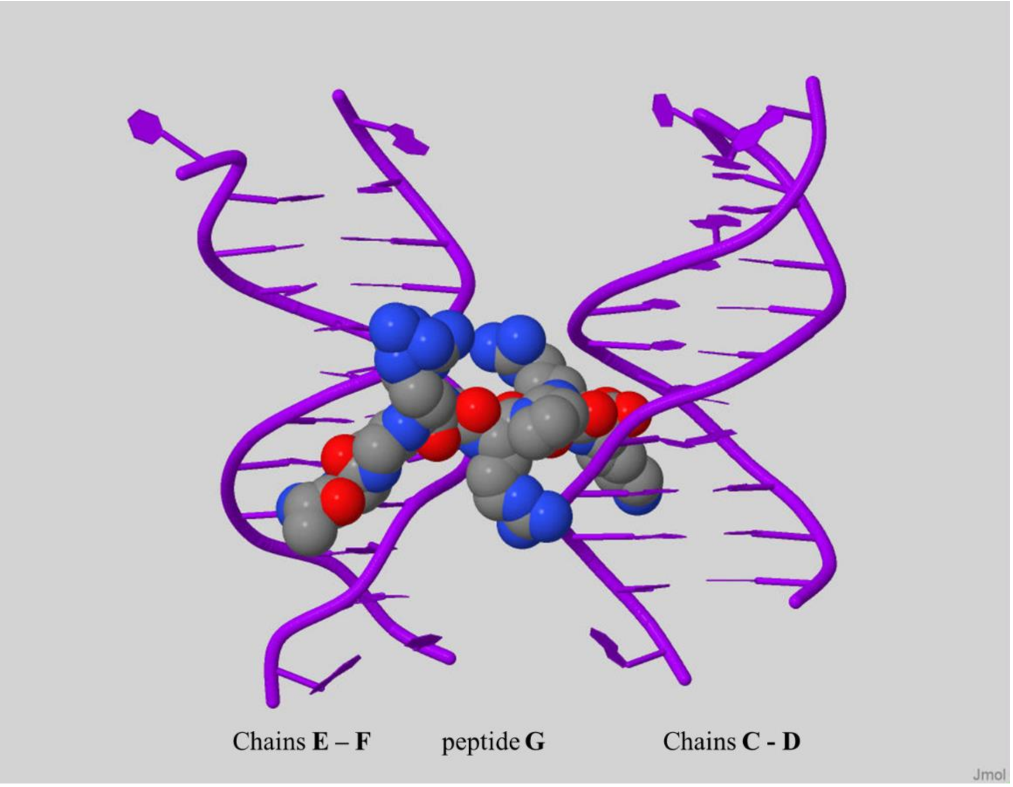

Figure S3. Peptide G is crosslinking Chains C-D and E-F in the A/T zone. The end of Chains shows that Guanine and Cytosine move out of the duplex conformation.

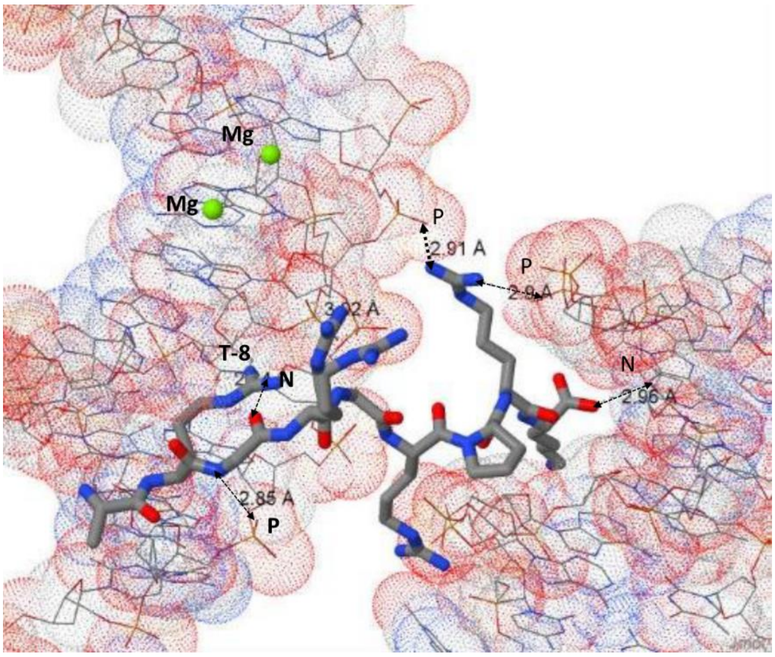

Figure S4. Cross-linking distances of Peptide G with Chains CD and EF**.**

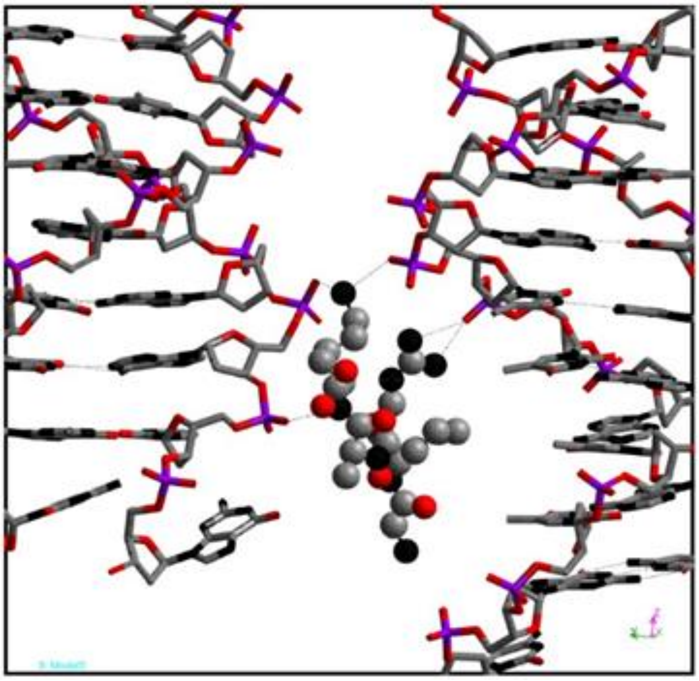

Figure S5. Peptide I is partially ordered and the amino acids 6-10 (GRPRK-NH_2) are visible in the structure interacting with chains E-F and chains A-B.

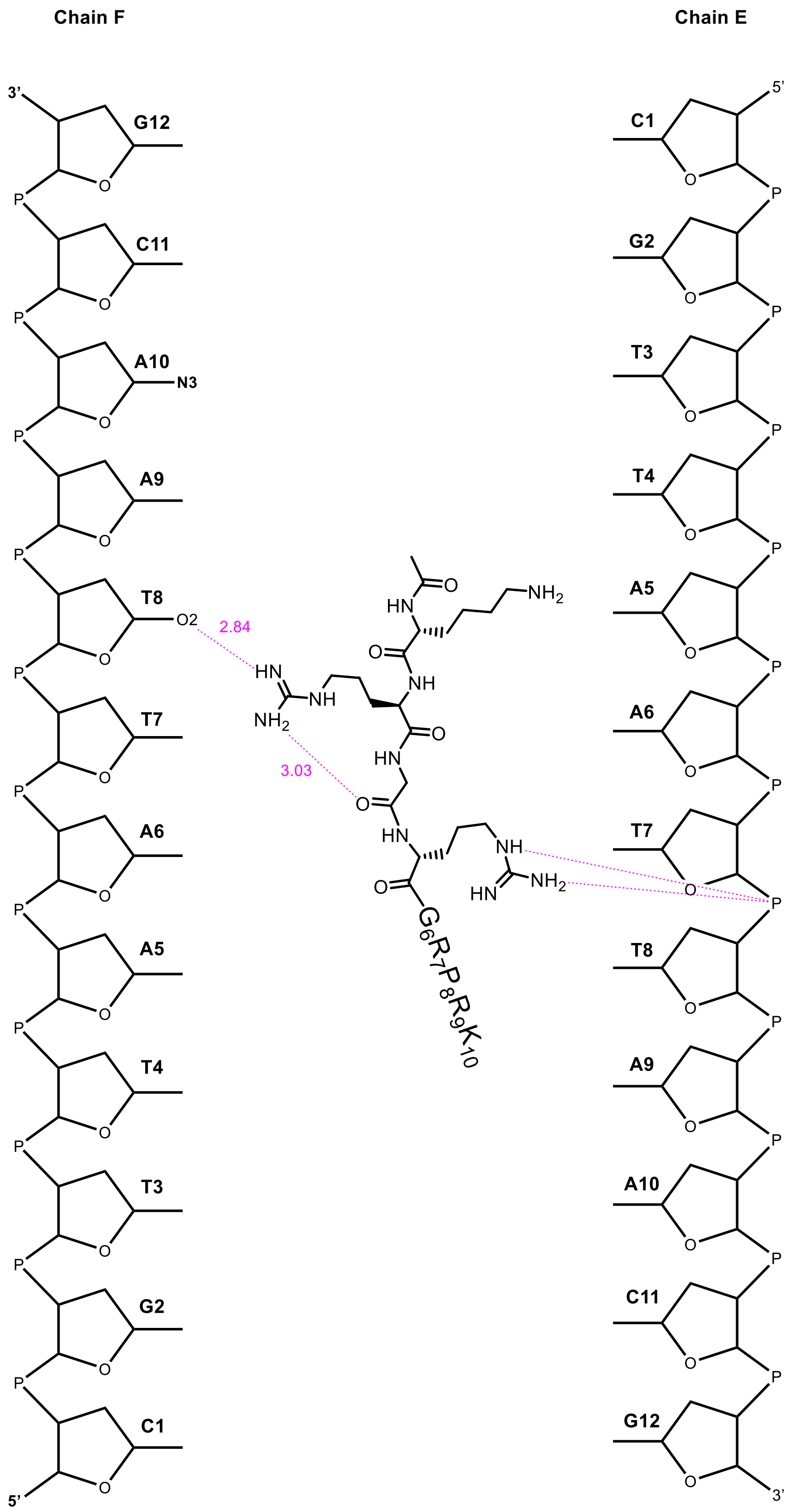

Figure S6. Schematic representation of interactions between peptide G with EF chains in the 8CPG structure. The $G_6R_7P_8K_{10}$ amino acids residues interacts with neighboring DNA (not shown).

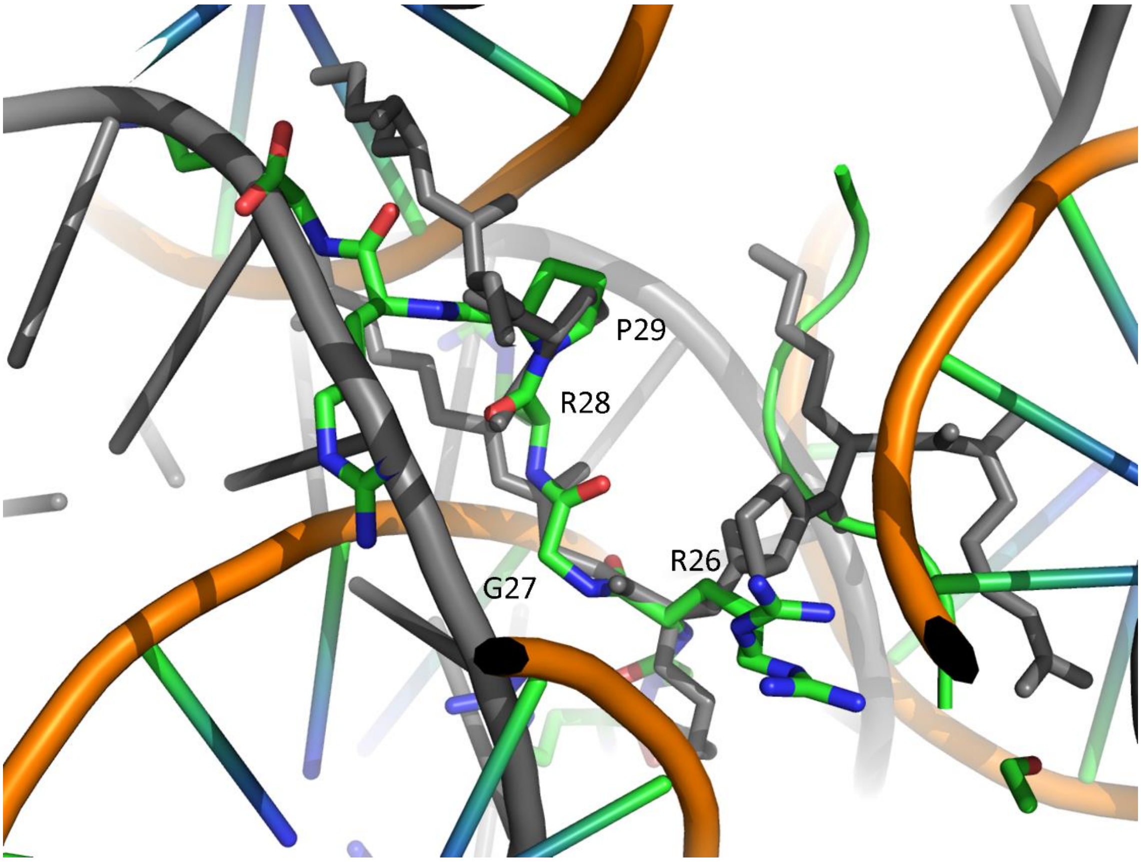

Figure S7. Overlapping of the PRGRP motifs common to AT-hook 1 (grey) and AT-hook 3 (coloured). The AT-hook 1 amino acid residues are numbered according to human HMGA1a protein (see Figure 1-A). The overall orientation of Pro29, Arg28, Gly27 and Arg26 is similar, with small local differences. In particular the conformation of the chain next to the central glycine is different. In the X-ray structure, AT-hook-3 enters more deeply into the minor groove and has a different orientation of the main chain NH groups, which form hydrogen bonds with thymines in the DNA. An additional difference is the position of the guanidinium group of Arg26, which is not uniquely positioned in the X-ray structure.

Molecular docking studies

Molecular docking studies were carried out using the Glide module of the Schrödinger's Maestro software (v12.9).[1] The 3-D crystallographic structure of B-DNA d(CGTTAATTAACG)$_2$ (PDB ID: 8CPG) was used as template for the docking studies. The PDB file for DNA-ligand complex was first prepared by removing the ligand (8CPG), solvent, salts and metal ions. The prepared DNA structure was further processed through "protein preparation wizard'' (Maestro wizard v12.9). The processing of the receptor grid followed the completion of the optimization process to calculate the binding pocket of the DNA. The coordinates of the receptor grid in the xyz Cartesian system are as follows: 8CPG (20.33, 37.40, 40.23). These coordinates indicate the enclosing box where ligand molecules are free to bind to DNA. Ligands were processed through "ligand preparation" tool. The ligands (**1**–**3**) were prepared using OPLS4 All-atom force field.[2] The docking studies were performed with standard precision and flexible ligand sampling.

Various docked ligand conformations were observed in the docking results, ranked by their binding energy scores and relative glide energy. Ranking based on scores provides a high rank for lesser scoring conformation.[2]

1. Docking of compounds 1–3 with the duplex d(CGTTAATTAACG)$_2$ (PDB: 8CPG)

In this experiment, we used the crystal structure reported here of the duplex d(CGTTAATTAACG)$_2$ (PDB: 8CPG) crystallised with AT-hook 1 of HMGA1. The interactions between the ligands (compounds **1**–**3**) and the duplex sequence d(CGTTAATTAACG)$_2$ were evaluated.

Table S1. Main H-bonding interactions observed by molecular docking of **1–3** with the 8CPG structure.

	Dihedral angles (º)			H-Bonds (Å)		
Cmpd	**N6-C-N5-C**	**C-N10-CO-C**	**N7’-C-N5’-C**			
**1**	174.6[a]	170.9	177.7	**NH6’-A10_N3**	**NH5-T8_O (PO_4)**	**NH6- T8_O (PO_4)**
				2.76	2.40	2.13
**2**	166.3	178.3	169.9		**NH5- T8_O (PO_4)**	**NH6-A9_ O (PO_4)**
					2.08	2.11
**3**	148.9	169.1	179.5	**NH6’- A5_N3**	**NH6’- A6_N3**	**NH6- T8_O (PO_4)**
				2.57	2.11	2.0

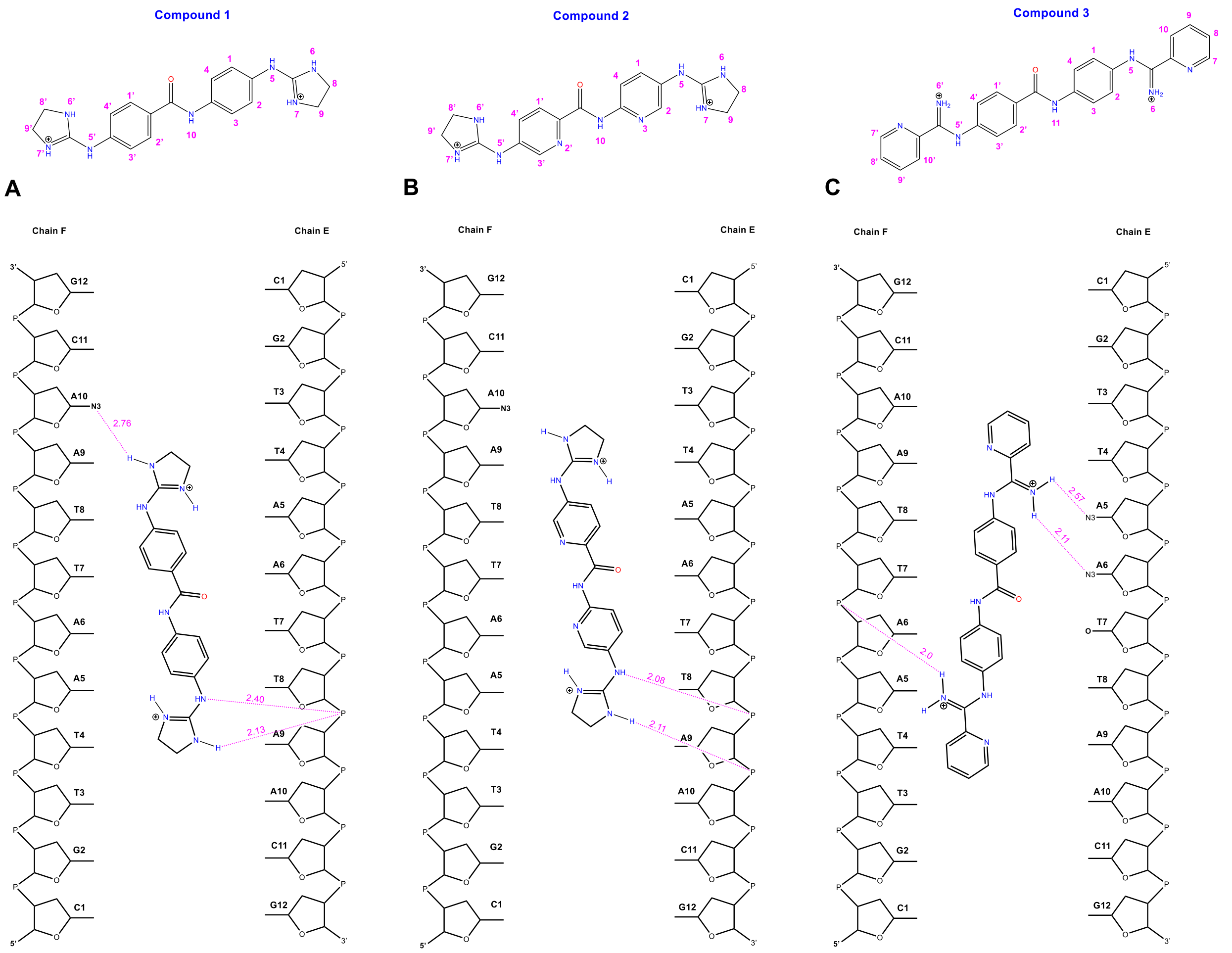

Figure S8. Schematic representation of interactions between compounds **1** (A), **2** (B) and **3** (C) with duplex **d1**_(TTAA)$_2$ as observed by molecular docking with 8CPG structure.

Table S2. Dihedral angles of optimized structures calculated with the B3LYP/6-31G(d) basis set using Gaussian 16 Rev. A.03.

	Dihedral angles (º)		
Cmpd	**N6-C-N5-C**	**C-N10-CO-C**	**N7’-C-N5’-C**
**1**	175.5	176.3	176.6
**2**	177.7	177.3	179.3
	**N6-C-N5-C**	**C-N11-CO-C**	**N7’-C-N5’-C**
**3**	162.4	178.6	162.8

Compound 1

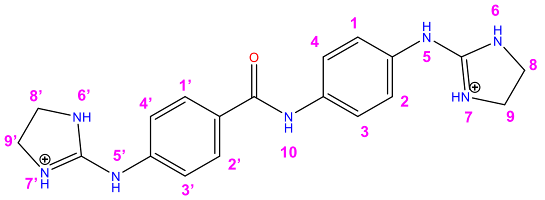

Compound 2

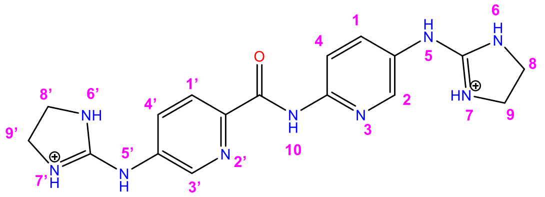

Compound 3

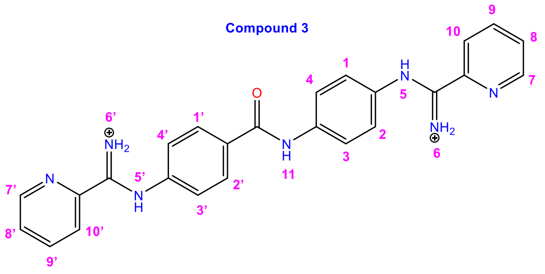

In vitro activity of compounds 1–3 against *T. brucei*, *T. cruzi* and *L. donovani*

Table S3. Activity (EC_{50}) of compounds **1**–**3** against *T. brucei*, *T. cruzi* and *L. donovani* and cytotoxicity (CC_{50}) against mammalian cells. Values (µM) are given as mean of at least 3 independent determinations and SEM (data taken from reference 3).

Cmpd	*T. brucei*[a]	HEK	SI[b]	*T. cruzi*[c]	L929	SI[d]	*L. donovani*[e]	THP-1	SI[f]
**1**	0.83 ± 0.08	>200	>240	>40	>200	nd	4.3 ± 0.54	nd	nd
**2**	25.5 ± 3.2	>200	>7.8	>40	>200	nd	>50	nd	nd
**3**	0.40 ± 0.02	>200	>500	0.21 ± 0.02<br>1.28 ± 0.34[g]	89.1 ± 8.3	424<br>69.6	0.26 ± 0.05<br>0.65 ± 0.20[h]	>50	>192<br>>76.9

[a] Trypomastigotes of *T. b. brucei* wild-type strain 427; Control drug: diminazene = 0.010 ± 0.0007 µM. [b] Selectivity index vs human endothelial kidney cells = CC_{50}/EC_{50} (*T. brucei*). [c] Epimastigotes of *T. cruzi* strain CL-B5 lacZ (DTU TcVI); control drug: benznidazole, EC_{50} = 25.3 ± 2.1 µM. [d] Selectivity index vs L929 fibroblasts = CC_{50}/EC_{50} (*T. cruzi*). [e] Promastigotes of *L. donovani* HU3; control drug: amphotericin B, EC_{50} = 0.07 ± 0.01 µM. [f] Selectivity index vs THP-1 cells = CC_{50}/EC_{50} (*L. donovani*). [g] Intracellular amastigotes of *T. cruzi* strain CL-B5 lacZ; benznidazole, EC_{50} = 0.54 ± 0.1 µM. [h]Intracellular amastigotes of *L. donovani* strain HU3; amphotericin B, EC_{50} = 0.19 ± 0.05 µM.

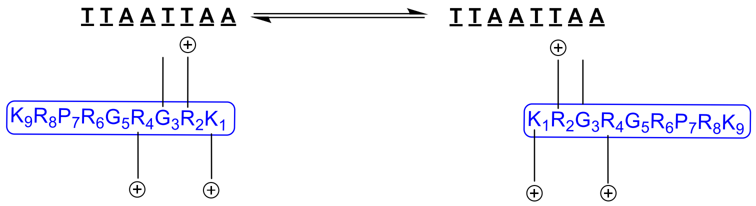

Figure S9. Schematic of the putative binding model of AT-hook 1 with $(TTAA)_2$ duplex in solution.

References

(1) *Maestro v12.9. Protein Preparation Wizard*; Schrödinger LLC: New York, NY, USA, 2012. (accessed.

(2) Jorgensen, W. L.; Maxwell, D. S.; Tirado-Rives, J. Development and Testing of the OPLS All-Atom Force Field on Conformational Energetics and Properties of Organic Liquids. *J. Am. Chem. Soc.* **1996**, *118* (45), 11225-11236. DOI: 10.1021/ja9621760.

(3) Nué-Martinez, J. J.; Cisneros, D.; Moreno-Blázquez, M. d. V.; Fonseca-Berzal, C.; Manzano, J. I.; Kraeutler, D.; Ungogo, M. A.; Aloraini, M. A.; Elati, H. A. A.; Ibáñez-Escribano, A.; et al. Synthesis and Biophysical and Biological Studies of N-Phenylbenzamide Derivatives Targeting Kinetoplastid Parasites. *J. Med. Chem.* **2023**, *66* (19), 13452-13480. DOI: 10.1021/acs.jmedchem.3c00697.
